# Xihuang pills targeting the Warburg effect through inhibition of the Wnt/β-catenin pathway in prostate cancer

**DOI:** 10.1016/j.heliyon.2024.e32914

**Published:** 2024-06-15

**Authors:** Fengxia Lin, Yan Long, Mingyue Li, Changlong Cai, Yongrong Wu, Xujun You, Xuefei Tian, Qing Zhou

**Affiliations:** a Department of Andrology, The First Affiliated Hospital of Hunan University of Chinese Medicine, Changsha, 410007, Hunan Province, China; b Department of Cardiovascular, Shenzhen Bao'an Chinese Medicine Hospital, Guangzhou University of Chinese Medicine, Shenzhen, 518000, Guangdong Province, China; c Graduate School of Hunan University of Chinese Medicine, Changsha, 410208, Hunan Province, China; d Department of Pharmacy, Shenzhen Bao'an Chinese Medicine Hospital, Guangzhou University of Chinese Medicine, Shenzhen, 518000, Guangdong Province, China; e College of Integrated Chinese and Western Medicine, Hunan University of Chinese Medicine, Changsha, 410208, Hunan Province, China; f Department of Urology, Shenzhen Bao'an Chinese Medicine Hospital, Guangzhou University of Chinese Medicine, Shenzhen, 518000, Guangdong Province, China; g School of Traditional Chinese Medicine, Hunan University of Chinese Medicine, Changsha, 410208, Hunan Province, China; h Department of Andrology, Shenzhen Bao'an Chinese Medicine Hospital, Guangzhou University of Chinese Medicine, Shenzhen, 518000, Guangdong Province, China

**Keywords:** Prostate cancer, Xihuang pills, Warburg effect, Wnt/β-catenin pathway, Metabolic re-programming

## Abstract

**Objective:**

Prostate cancer, marked by a high incidence and mortality rate, presents a significant challenge, especially in the context of castration-resistant prostate cancer (CRPC) with limited treatment options due to drug resistance. This study aims to explore the anti-tumor effects of Xihuang Pills (XHP) on CRPC, focusing on metabolic reprogramming and the Wnt/β-catenin pathway.

**Methods:**

In vitro and in vivo biofunctional assays were employed to assess the efficacy and mechanisms of XHP. Subcutaneous xenografts of PC3 in mice served as an in vivo model to evaluate XHP's anti-tumor activity. Tumor volume, weight, proliferation, and apoptosis were monitored. Various assays, including CCK8, TUNEL assay, QRT-PCR, and Western Blotting, were conducted to measure metabolic reprogramming, proliferation, apoptosis, and cell cycle in prostate cancer cells. RNA-seq analysis predicted XHP's impact on prostate cancer, validating the expression of Wnt/β-catenin-related proteins and mRNA. Additionally, 58 compounds in XHP were identified via LC-MS/MS, and molecular docking analysis connected these compounds to key genes.

**Results:**

In vitro and in vivo experiments demonstrated that XHP significantly inhibited CRPC cell viability, induced apoptosis, and suppressed invasion and migration. mRNA sequencing revealed differentially expressed genes, with functional enrichment analysis indicating modulation of key biological processes. XHP treatment downregulated Wnt signaling pathway-related genes, including CCND2, PRKCG, and CCN4. Moreover, XHP effectively inhibited glucose uptake and lactate production, leading to reduced HIF-1α and glycolytic enzymes (GLUT1, HK2, PKM2), suggesting its potential in attenuating the Warburg effect. Molecular docking analysis suggested a plausible interaction between XHP's active compounds and Wnt1 protein, indicating a mechanism through which XHP modulates the Wnt/β-catenin pathway.

**Conclusion:**

XHP demonstrated remarkable efficacy in suppressing the growth, proliferation, apoptosis, migration, and invasiveness of prostate tumors. The interaction between XHP's active constituents and Wnt1 was evident, leading to the inhibition of Wnt1 and downstream anti-carcinogenic factors, thereby influencing the β-catenin/HIF-1α-mediated glycolysis.

## Graphical abstract

**Figure:**
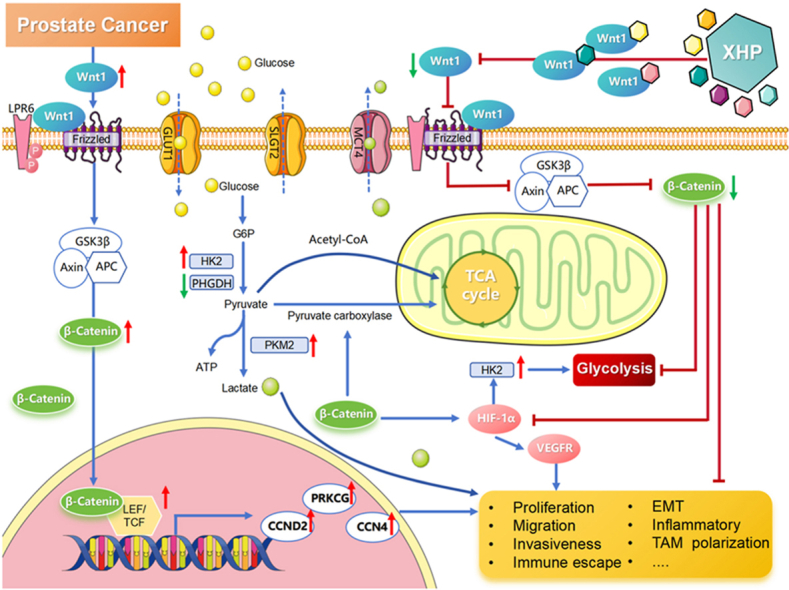

## 1 Introduction

Prostate cancer (PCa) presently holds the foremost position in incidence and ranks second in mortality among male malignant tumors. It has emerged as a significant public health concern, posing a serious threat to male health [1]. The increasing global aging population has contributed to a rise in the incidence of prostate cancer. While surgery, radiotherapy, and androgen deprivation therapy demonstrate efficacy in managing early-stage prostate cancer, the challenge lies in the detection of some cases only during advanced stages. This is attributed to the relatively inconspicuous clinical symptoms associated with early prostate cancer [2]. Late-stage localized prostate cancer invariably progresses to castration-resistant prostate cancer (CRPC), characterized by heightened metastasis, invasion, and resistance. This advanced stage carries a significantly poor prognosis [3]. Conventional chemotherapy exhibits limited sensitivity and specificity for CRPC, often resulting in substantial side effects. This is particularly problematic for patients with comorbidities and those in the elderly demographic [4]. While second-generation antiandrogens (SGAs), such as abiraterone, enzalutamide, apalutamide, and darolutamide, have extended the overall survival time for CRPC patients, a notable 20–40 % of individuals do not derive significant benefits from these treatments [5,6]. Therefore, finding or developing new drugs to treat CRPC is imperative.

The anti-tumor effects of traditional Chinese medicine and its effective ingredients are increasingly attracting attention. Xihuang Pills (XHP), a Chinese herbal formula recorded in ancient books more than 280 years ago, is composed of four traditional Chinese medicines: *Calculus Bovis (from Bos taurus domesticus Gmelin), Moschus (from Moschus berezovskii Flerov), Olibanum (Boswellia carterii Birdw.), and Myrrha (from Commiphora myrrha Engl.)*. Recently, XHP have been widely used in the treatment of malignant tumors and have shown good therapeutic effects [7]. Multiple meta-analyses studies have shown the clinical efficacy and safety of XHP in the treatment of breast cancer [8], gastric cancer [9], and lung cancer [10]. XHP demonstrate the ability to induce apoptosis in tumor cells, inhibit their proliferation, regulate the tumor microenvironment, modulate autophagy levels, and influence tumor immunity and macrophage polarization. These multifaceted actions position XHP as a potential therapeutic agent in the treatment of various malignant tumors [11]. In our previous work, we identified 37 active components of XHP and demonstrated their in vitro and in vivo therapeutic effects in prostate cancer [12]. With the increasing number of studies showing that changes in metabolic patterns lead to the progression of CRPC [13], it is of great significance to explore the role of XHP in re-programming prostate cancer metabolism and the related mechanisms.

Metabolic reprogramming plays a pivotal role in the progression of prostate cancer and the development of treatment resistance. In prostate cancer, this reprogramming entails a shift from oxidative phosphorylation to aerobic glycolysis, fostering the production of energy and essential biomolecules crucial for tumor growth and survival [14]. This characteristic, commonly referred to as the Warburg effect, is a phenomenon consistently observed in various tumor types. It is intricately linked to the initiation and progression of cancer [15]. It entails the conversion of glucose into lactic acid by tumor cells, even under sufficient oxygen conditions. Over the past decade, there has been a growing interest in targeting crucial steps of aerobic glycolysis as a promising avenue in cancer research [16]. Various enzymatic inhibitors aimed at essential components of glucose metabolism [17] have been tested in clinical trials, although their routine clinical application remains uncertain. The promotion of increased aerobic glycolysis is associated with the growth of various cancers, thus there is a need to develop new therapeutic approaches to prevent metabolic reprogramming in cells. Traditional Chinese medicine, exemplified by XHP, holds promise as a therapeutic agent against the initiation, promotion, and advancement of cancer through diverse mechanisms. The modulation of cellular energy metabolism stands as a compelling focus in cancer research, necessitating innovative therapeutic strategies, as highlighted earlier. This study, delving into the potential of XHP in addressing CRPC, introduces novel insights into the applicability of metabolic reprogramming as a viable avenue for cancer treatment. The encouraging outcomes of this research lay the groundwork for future investigations into the role of XHP in prostate cancer treatment, fostering optimism for enhanced outcomes in individuals with CRPC.

## 2 METHODS

### 2.1 Reagents and antibodies

XHP was purchased from Tong Ren Tang (z11020073, Beijing, China). DMEM was purchased from Hyclone (SH30243.01, Logan, USA). RMPI-1640 was purchased from Hyclone (SH30809.01 B, Logan, USA). CCK-8 was purchased from Signalway Antibody (CP002, MA, USA). Fetal bovine serum was purchased from GIBCO (16000–044, NY, USA) Trypsin-EDTA (0.25 %) was purchased from Solarbio(T1300-100, Beijing, China). Matrigel was purchased from Corning (356234, NY, USA). 2-NBDG Glucose Uptake Assay Kit was purchased from Abcam (ab235976, Cambridge, UK). Penicillin/streptomycin was purchased from Solarbio(P1400-100, Beijing, China). Lactic Acid Assay kit was purchased from Nanjing Jiancheng Bioengineering Institute (A109-2, Jiangsu, China). Trizol was purchased from Invitrogen (1596–026, CA, USA). SYBR Green PCR Kit was purchased from Thermo (#K0223, MA, USA). Reverse Transcription Kit was purchased from Thermo (#K1622, MA, USA). Annexin-V-FITC was purchased from Biomics Biotechnologies (C1062, Beijing, China). RNase A was purchased from Solarbio (R8020-25, Beijing, China). TUNEL Assay Kit was purchased from Roche (11684817910, Basel, Switzerland). The antibodies used in this study was purchased from Abcam(Cambridge, UK) include: HIF1a(ab179483); GULT1(ab115730); HK2(ab209847); PKM2(ab85555); Wnt1(ab15251); β-Catenin(ab32572). GAPDH was purchased from Proteintech(60004-1-Ig, Chicago, USA). The secondary anti-IgG(HRP conjugated-goat anti-mouse IgG) was purchased from ZSGB-BIO(ZB-2305, Beijing, China).

### 2.2 Animals

Male Sprague-Dawley rats(6–8 weeks) with a body mass of 220 g ± 40 g and male BABL/c nude mice (4–6 weeks) with a body mass of 22 g±2 g were procured from the Shenzhen Glorbay Biotech Co.,Ltd.(Guangdong, China). The experiments adhered to approved guidelines and were sanctioned by the ethics committee of Shenzhen Glorybay Biotech Laboratory (No. RW-IACUC-22-0025).

### 2.3 Cell lines and culture conditions

For our standard experiments, we employed the docetaxel-resistant, androgen-independent human prostate cancer cell lines PC-3 and DU145, both of which were found to be negative for AR-FL and AR-V7. These cell lines were procured from ATCC (VA, USA). To ensure precise monitoring of subcutaneous tumor growth using IVIS spectrum, we chose to inoculate PC-3-Red-Fluc cells obtained from PerkinElmer (MA, USA). All the cells were cultured at 37°Cwith 5 % (v/v) CO_2_ in a controlled humid environment, using RPMI-1640 medium supplemented with 10 % fetal bovine serum and 1 % penicillin/streptomycin.

### 2.4 Qualitative LC-MS/MS analysis of XHP

As mentioned in the previous work, Liquid Chromatography-Tandem Mass Spectrometry(LC-MS/MS) method was utilized to qualitatively determine the constituents of XHP. After accurately weighing 2 g of Xihuang pill powder, it was dissolved in 20 ml of methanol, filtered through a 0.22 μm membrane filter, and then centrifuged at 8000×*g* for 5min. The resulting supernatant was collected for analysis. The mass spectrometric analysis was conducted using the ESI negative and positive-ion mode of the Agilent 1290UPLC-6540-Q-TOF accurate mass spectrometer system equipped with an ESI ion source (Agilent Technologies, Santa Clara, CA, USA). The operating parameters were set according to the previous work.

### 2.5 Preparation of XHP extract and drug-containing serum

The XHP powder (3 g) was dissolved in 6 ml of double distilled water for a duration of 24 h. Subsequently, the solution underwent ultrasonic treatment for 2 min before being centrifuged at 6000 rpm for 10 min. The resulting supernatant and sediment were both collected. The sediment was then dissolved in 6 ml of DMSO using an ultrasound device and subjected to a second centrifugation at 6000 rpm for 10 min. By mixing the supernatant obtained from the initial and subsequent centrifugation, an XHP solution with a final concentration of 500 mg/ml was prepared. The resulting solution was stored at 4 °C for later use.

For adult individuals, the prescribed amount of XHP per day is 6 g, which translates to 62.5 mg/kg for rats. The dose calculation takes into account the variance in body surface area between humans and rats. To perform the animal study, a suspension of XHP extract at a concentration of 6.25 mg/ml was prepared by dissolving it in DMSO. The rats were orally administered Xihuang pill at a dose of 10 ml/kg once daily for 14 consecutive days. Subsequently, 4 h after the final dose, blood samples were collected from the abdominal aorta of the rats via intraperitoneal injection of chloral hydrate. After allowing it to stand at room temperature for 2 h, the serum was separated by centrifugation and subjected to a water bath at 56 °C for 30 min. Finally, the serum was sterilized using a 0.22-μm microporous filter and stored at a temperature of −80 °C.

### 2.6 Cell viability assay

PC3 and DU145 cells were seeded at a density of 5000 cells per well in 96-well plates and treated with varying concentrations of XHP Extract or drug-containing serum for 48 h. We conducted preliminary experiments to explore the effect of XHP extract and drug-containing serum on the viability of PC3 cells (see Supplementary Material 1). Finally, we selected 10 % XHP drug-containing serum as the concentration for subsequent experiments. Afterwards, 10 μl of Cell Counting Kit-8 (CCK-8) solution was added to each well and the cells were incubated at 37°Cwith 5 % CO_2_ for 2 h. The viability of the cells was assessed by measuring the absorbance at 450 nm using a microplate reader (DNM-9602, Perlong).

### 2.7 Cell cycle analysis

PC3 cells and DU145 cells were cultured in T25 flasks and exposed to 10 % XHP drug-containing serum or blank serum at 37 °C. The cells were collected, treated with ethanol to fixate them, and then exposed to ribonuclease before being stained with propidium iodide. The resulting DNA content was analyzed using a FACScan flow cytometer(Accuri C6, BD).

### 2.8 Flow cytometry for cell apoptosis

PC3 cells and DU145 cells were seeded in a T25 culture flask and incubated with either 10 % XHP drug-containing serum or blank serum at 37 °C for 24 h. Next, the cells were harvested and fixed with precooled 4 % paraformaldehyde. Following a PBS wash, the fixed cells were treated with 0.25 % trypsin (EDTA excluded) and centrifuged at 1000 rpm for 5 min. The collected cells were then washed again with PBS and suspended in 500 μl binding buffer. To this mixture, 5 μl of propidium iodide and Annexin V-APC were added, and the whole was kept in the dark at room temperature for 5–15 min. Apoptosis analysis was conducted using a Canto II flow cytometer (Accuri C6, BD).

### 2.9 Transwell assay

#### 2.9.1 Transwell Invasion assay

The pre-cooled transwell chamber of 24-well format transwell plates was coated with 80 μL of a 1:2 dilution of matrigel per well on ice. PC3 cells and DU145 cells were seeded in the upper chamber at a density of 3 × 10^5^ cells/well in 200 μL of medium and treated with 10 % XHP drug-containing serum or blank serum. The lower chamber contained 700 μL of medium containing 10 % FBS. After 48 h, the cells were fixed in 4 % buffered formalin and stained with 0.5 % crystal violet (0528, Solarbio, China). The cells on the upper side of the filter were removed with a cotton swab. The staining images were observed and recorded using a phase-contrast fluorescent microscope (CX41RF, Olympus) with a magnification of × 200.

To further assess whether Xihuang Pills (XHP) can reverse the invasion ability of PC3 under hypoxic conditions, we subjected the cells to a hypoxic environment by exposing them to a gas mixture containing 1 % oxygen, 5 % carbon dioxide, and the remaining nitrogen. XHP medicated serum and blank (control) medicated serum were subsequently added at the targeted concentrations. The Transwell Invasion Assay was then repeated as described above to evaluate whether Xihuang Pills exhibit anticancer effects under hypoxic conditions.

#### 2.9.2 Transwell migration assay

With the exception of the uncoated chamber and a reduced incubation time of 24 h, the methodology for the migration assay was identical to that of the invasion assay.

### 2.10 EdU

PC-3 cells were initially seeded into a 12-well plate and subjected to overnight incubation in a chamber maintained at 37 °C using the Innova CO-48 system (New Brunswick Scientific, Edison, NJ, USA). Subsequently, the cells were exposed to hypoxia in a gas mixture equilibrated with 1 % O_2_, 5 % CO_2_, and the remaining nitrogen. Following this, Xihuang Pills (XHP) containing medicated serum and control serum were sequentially added at the target concentrations. An EdU working solution was then prepared and added to the 12-well plate, and the cells were incubated for an additional 2 h. The culture medium was discarded, and cells were fixed with 4 % paraformaldehyde at room temperature for 15 min. After fixation, the fixing solution was removed, and cells were washed three times with PBS. Cell membranes were permeabilized with 0.25 % Triton X-100 at room temperature for 10 min. Subsequently, the permeabilization solution was removed, and cells were washed twice with PBS. The Click reaction solution was prepared, and each well was treated with 0.5 ml of the Click reaction solution, followed by a dark incubation for 30 min. After incubation, cells were washed three times with PBS. Finally, cell nuclei were stained with DAPI, and EdU signals were measured using fluorescence microscopy, with images captured and saved for data analysis. This series of steps aims to systematically evaluate the impact of Xihuang Pills on PC-3 cells under hypoxic conditions, with a particular focus on its effects on cell proliferation.

### 2.11 Glucose uptake detection

PC3 cells and DU145 cells were initially plated at a density of 1 × 10^5^ cells per well in a 6-well plate and left to adhere for 24 h. Subsequently, the cells were treated with either 10 % XHP drug-containing serum or blank serum in glucose-free medium for 48 h. Ten minutes before the end of the treatment, add 2-NBDG to a final concentration of 200 μg/ml in glucose-free medium. A FACScan flow cytometer (Accuri C6, BD) was utilized for glucose uptake detection. To assess whether XHP can regulate the glycolysis of PC3 cells under hypoxic conditions, we exposed PC-3 cells to a hypoxic environment with 1 % oxygen, as described earlier. Similarly, after a 48-h intervention with 10 % XHP drug-containing serum or blank serum, we employed the same method to detect glucose uptake. To further appraise the impact of Xihuang Pills (XHP) on normal prostate cells, we conducted a repetition of the aforementioned glucose uptake assay using the RWPE-1 cell line, which serves as a representative model for normal prostate epithelial cells.

### 2.12 Lactate detection

PC3 and DU145 cells were plated at a density of 1 × 10^5^ cells per well in a 6-well plate and allowed to adhere for 24 h. Next, the cells were exposed to either 10 % XHP-containing serum or blank serum in glucose-depleted medium for 72 h. The lactate concentrations in the media were monitored over the 72-h period using the Lactic Acid Assay kit.

### 2.13 Animal treatment

After a one-week acclimatization period, PC-3-Red-Fluc cells (1.0 × 10^7^ cells in 200 μL PBS per mouse) were subcutaneously inoculated into the mice. When the tumor reached a volume of 20mm3, the nude mice were randomly divided into two groups: the control group and the XHP group. In adherence to the 4 R principles (reduction, refinement, replacement, responsibility), we conscientiously considered ethical and welfare aspects in the design of our animal experiments. Striving to minimize the number of animals used while maximizing statistical significance, we applied the Reduction principle to allocate eight mice per group for both the control group and the XHP group. The XHP group was given a daily dose of 78 mg/kg for 3 weeks based on the surface area ratio conversion between humans and mice, while the control group received an equivalent volume of normal saline. After undergoing continuous inhalation of isoflurane anesthesia via the XGI-8 small animal anesthesia apparatus(Xengoen, USA), the size of the tumors in the nude mice was monitored every three days through the detection of fluorescent radiation by the IVIS Spectrum(Xengoen, USA). Selected for near-infrared red fluorescence imaging, with excitation and emission wavelengths of 328 and 533 nm, a visual field of 22.2 × 22.2 cm^2^, an aperture of f/2.0, and a 1-s exposure time. The tumor's volume was computed using the formula: volume (mm3) = 1/2 × length × width2. Three weeks after drug administration, the mice were euthanized, and tumor tissues were harvested for further analysis.

### 2.14 Measurement of glucose and lactic acid in tumor

Each subcutaneous tumor was dissected and 10 mg of tissue was collected. The tissue was then digested using 10 mg of Type I Collagenase (49E19342, Worthington, USA) at 37 °C for 60 min. The resulting digest was centrifuged at 1200 rpm for 5 min. The levels of glucose and lactic acid were measured using the 2-NBDG Glucose Uptake Assay Kit and the Lactic Acid Assay kit, respectively.

### 2.15 Hematoxylin and eosin (H&E) staining

The tissue samples were fixed in a solution of 10 % neutral-buffered formalin for 48 h and subsequently embedded in paraffin wax. The paraffin-embedded tissues were sectioned into 4-μm slices, and then deparaffinized, rehydrated, and rinsed. The H&E staining was conducted to assess the pathological change of tumor tissues according to the procedure described previously. And the images were captured with microscope (CX41RF, Olympus).

### 2.16 Evaluation of tumor cell apoptosis in vivo

TUNEL assay was carried out using the TUNEL Assay Kit in accordance with the manufacturer's instructions. Specifically, the tissue sections were treated with proteinase K and the TUNEL reaction mixture, followed by counterstaining with DAPI and mounting with coverslips. Fluorescence microscopy was employed to capture images of the stained tissue sections, utilizing appropriate filters for both fluorescein and DAPI.

### 2.17 Immunohistochemistry staining

The specimens were subjected to incubation at a temperature of 4 °C for an entire night, using Ki67 (1:200). Subsequently, they were incubated with a goat antirabbit secondary antibody for 60 min at a temperature of 37 °C, and were visualized using diaminobenzidine (DAB). The cellular nuclei were then stained with hematoxylin for a duration of 3 min at 25 °C. Representative tissue images were captured using a light microscope and the positive regions were analyzed using the Image-Pro Plus 6.0 software.

### 2.18 Reverse transcription and quantitative polymerase chain reaction (RT-qPCR)

The total of the RNA was extracted via the utilization of TRIzol reagent. Subsequently, the extracted RNA samples underwent reverse transcription through employment of the Reverse Transcription Kit. In duplicate, qPCR was executed on the samples using 2X SYBR Green qPCR Master Mix, as per the manufacturer's instructions, and assessed through implementation of the 2−ΔΔCt method. The expression levels of the target genes were evaluated after normalizing the mRNA amounts against endogenous GAPDH. Primer sequences are shown in Supplementary Material 2.

### 2.19 Western blot (WB)

The RIPA lysate was employed to lyse cells or mouse tumor tissues for extracting the total protein, which was then quantified using the BCA protein quantitative kit. Subsequently, the protein samples underwent separation via sodium dodecyl sulfate-polyacrylamide gel electrophoresis (SDS-PAGE), followed by transfer onto a polyvinylidene fluoride (PVDF) membrane activated with methanol and sealed using 5 % skim milk, and subsequently dried at room temperature for at least an hour. Following this, the membrane was incubated overnight at 4 °C with the primary antibody, and then washed thrice in 1x TBST for 5 min each before being incubated with the horseradish peroxidase-conjugated secondary antibody at ambient temperature for an hour. Finally, the blots were visualized using Thermo Fisher Scientific's SuperSignal West Pico Stable Peroxide Solution.

### 2.20 Differential gene expression and enrichment analysis in prostate cancer

Total RNA was extracted using TRIzol reagent, and RNA concentration was measured using a NanoDropTM 2000. RNA integrity numbers were determined using an RNA 6000 Nano kit (5067–1511, CA, USA) purchased from Agilent Technologies. Library construction, quality control, and deep sequencing were performed by Allwegene Biotechnology Company (Shenzhen, China). Differential expression analysis between the two comparison groups was performed using DESeq software (1.20.0). DESeq was used for differential gene expression analysis, with screening criteria for differentially expressed genes set as: |log2FoldChange| > 1, and a significant P-value <0.05. TopGO was used for differential gene enrichment analysis in GO enrichment analysis, calculating P-values using the hypergeometric distribution method (significant enrichment criterion: P-value <0.05) to identify significantly enriched GO terms and determine the main biological functions of differentially expressed genes. The clusterProfiler (3.4.4) software was used for KEGG pathway enrichment analysis, focusing on significantly enriched pathways with a P-value <0.05.

### 2.21 Active components-targets docking

Based on the 58 compounds identified through LC-MS/MS, we downloaded the active compound mol2 format files from the PubChem database. Receptor files were prepared by downloading Wnt1 pdb format files from the Protein Data Bank (PDB) and converting them to pdbqt files using AutoDockTools. Molecular docking analysis was performed using AutoDock software to predict the binding affinity between proteins and compounds. It is generally believed that the lower the affinity between a receptor and a ligand, the greater the stability and the more successful the docking. We selected the molecule with the lowest binding energy in the docking conformation and observed the binding effect by matching the interactions between the ligand and the molecule.

### 2.22 Statistics and analysis

Experimental data comprising of 3 or more replicates were scrutinized for statistical significance across different experiments using one-way ANOVA followed by post hoc Tukey test, conducted with GraphPad Prism version 8.0.2 (GraphPad software, CA, USA). Results are presented as means ± SEM and were considered statistically significant (*) at p < 0.05.

## 3 RESULTS

### 3.1 Qualitative assessment of XHP' constituents

The components of XHP underwent qualitative analysis using LC-MS/MS. We identified 58 compounds in the XHP extract, including Kezitanin, D-9-AnthrylaAlanine, and Curcumone. Supplementary Material 3 provides a comprehensive list of the identified compounds and the chromatograms of total ion. The pivotal constituents within Xihuang Pills (XHP), notably Taurocholic acid and Abietic acid, are in concordance with the essential content specifications delineated in the Chinese Pharmacopeia (2020). This serves as compelling evidence affirming that the XHP sourced from Beijing Tong Ren Tang adheres to the prescribed quality standards.

### 3.2 Inhibition of tumor growth and induction of apoptosis in prostate cancer by XHP treatment in vivo

The mice were inoculated with subcutaneous xenografts of PC3 to investigate the in vivo anti-tumor activity of XHP. We monitored tumor size in the nude mice triweekly utilizing the IVIS Spectrum, as illustrated in Fig. 1 A-C. Treatment with a daily dose of 78 mg powder/kg/day XHP for three weeks significantly suppressed tumor growth. Additionally, no statistically significant differences in body weight were observed between the control and XHP groups. These findings indicate that XHP impedes tumor formation in prostate cancer-afflicted mice.

**Fig. 1:**
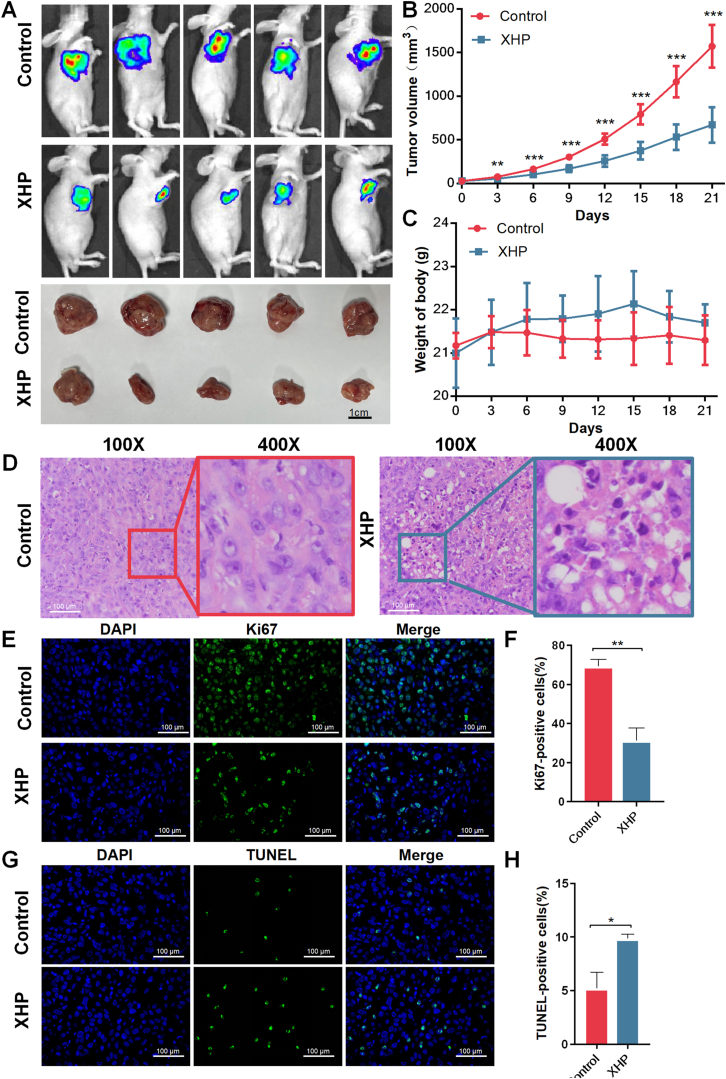
In vivo anti-tumor activity of XHP. (A) In vivo bioluminescence images and tumor physical map of tumors in each group after treatment; (B) Dynamic alteration of tumor volumes amid the therapeutic intervention; (C) Dynamic alteration of weight of body amid the therapeutic intervention; (D) Histological outcomes revealed by Hematoxylin and Eosin (H&E) staining in diverse PC3 prostate cancer subcutaneous graft scenarios; (E–F) Immunohistochemical evaluations depicting Ki67 staining divergence within PC3 prostate cancer subcutaneous grafts across distinct groups; (G–H) The TUNEL assay outcomes portraying apoptosis in PC3 prostate cancer subcutaneous grafts among different groups. XHP: Xihuang Pills. P value: *P < 0.05, **P < 0.01, ***P < 0.001.

Building upon this observation, we sought to examine the impact of XHP on pathological tissue in subcutaneously transplanted prostate cancer. We employed hematoxylin and eosin staining under a microscope to assess alterations in pathological tissue. Results revealed that in the control group, tumor cells exhibited robust growth, irregular morphology, disordered arrangement, large and intensely stained nuclei, substantial nuclear pleomorphism, numerous mitotic figures, scant stromal components, disrupted nucleocytoplasmic ratios, indistinct cell membranes, and hematopoietic processes between the beam and cable. In contrast, the XHP group displayed cell nuclei that diminished or fragmented, and apoptotic bodies formed (as shown in Fig. 1 D).

To further elucidate the underlying mechanisms of XHP's inhibitory effects, we evaluated its influence on proliferation and apoptosis in subcutaneously transplanted prostate cancer. We conducted immunofluorescence detection on the xenograft tumors, examining the proliferation-related marker Ki67. As depicted in Fig. 1E–F, XHP effectively suppressed the expression level of Ki67. To determine the effect of XHP on apoptosis, we employed TUNEL monitoring. Results demonstrated that the tumor cell apoptosis rate induced by XHP was 9.87 %, as opposed to the control group's 5.22 %, verifying XHP's capacity to induce apoptosis in prostate cancer cells(as shown in Fig. 1G–H).

### 3.3 In Vitro Antitumor Activity of XHP: inhibition of cell viability, induction of apoptosis, and suppression of invasion and migration in prostate cancer cells

To evaluate the in vitro antitumor activity of XHP, we prepared XHP-containing serum and XHP extracts. In preliminary experiments, we used the CCK-8 assay to assess the drug's effects on the viability of PC-3 cells, discovering that both XHP-containing serum and XHP extracts exhibited potent inhibitory effects on cell viability. To minimize the impact of nonspecific effects from the compound extract on the results, we selected XHP-containing serum as the intervention method for subsequent cell experiments. As depicted in Fig. 2A, compared to blank serum, XHP-containing serum demonstrated excellent concentration-dependent inhibition of cell viability.

**Fig. 2:**
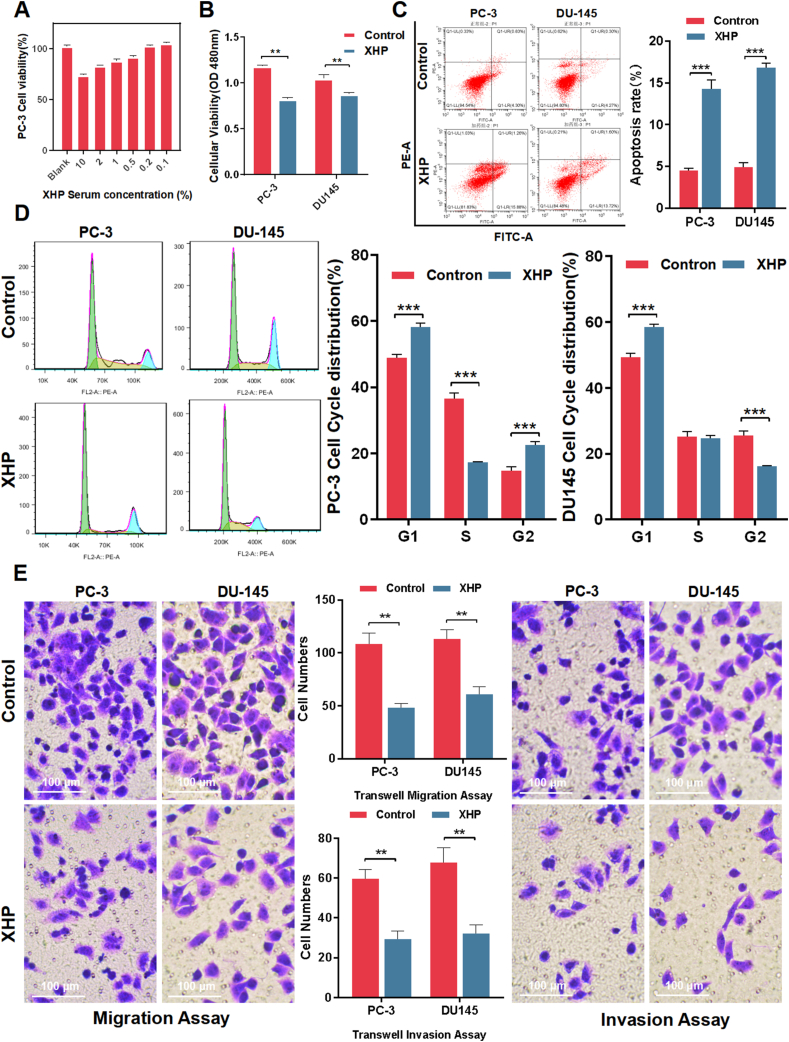
In Vitro Antitumor Activity of XHP. (A) Impact assessment of different concentrations of XHP-containing serum on PC-3 cell viability using CCK-8 assay; (B) CCK-8 assay evaluating the effects of 10 % XHP-containing serum on the viability of PC-3 and DU145 cells; (C) Assessment of apoptosis in PC-3 and DU145 cells treated with XHP-containing serum using Annexin V-APC staining; (D) Flow cytometry analysis of the influence of XHP-containing serum on the cell cycle of PC-3 and DU145 cells; (E) Evaluation of the effects of XHP-containing serum on the invasion and migration of PC-3 and DU145 cells using Transwell Invasion and Migration Assays. P value: *P < 0.05, **P < 0.01, ***P < 0.001.

Having established the inhibitory effects of XHP-containing serum on cell viability, we sought to investigate its impact on docetaxel-resistant, androgen-independent human prostate cancer cell lines. We employed a 10 % XHP-containing serum concentration to intervene with the PC-3 and DU145 cell lines. The observed inhibition rates were −30.22 % and −25.53 %, respectively (Fig. 2B), indicating that XHP displays notable efficacy in suppressing the viability of castration-resistant prostate cancer cells. Using Annexin V-APC staining and flow cytometry, we examined the changes in apoptosis and cell cycle in both cell lines 24 h after XHP treatment. As shown in Fig. 2C, compared to the control group, the apoptosis rate in the XHP group increased significantly. As illustrated in Fig. 2D, XHP effectively blocked the cell cycle transition from G1 to S phase, with a statistically significant difference compared to the control group. Furthermore, we employed Transwell Invasion and Migration Assays to investigate the influence of XHP on the invasive and migratory capabilities of prostate cancer cells. Results revealed that XHP significantly inhibited the invasive and migratory abilities of PC-3 and DU145 cells, displaying a statistically significant difference compared to the control group (Fig. 2E). In an environment where oxygen levels are restricted to 1 %, mimicking hypoxia, we expanded our assessment to discern the potential of XHP in alleviating the proliferation and invasion of prostate cancer cells. Remarkably, findings from the EdU experiment unveiled that, even amid hypoxic conditions, XHP manifested conspicuous inhibitory impacts on PC-3 cell proliferation. Furthermore, within the Transwell Invasion Assay, Xihuang Pills showcased a noteworthy suppression of invasive tendencies in PC3 cells under hypoxic condition (Supplementary Material 4).

### 3.4 Exploring the mechanism of XHP's anti-prostate cancer effects: mRNA sequencing and pathway analysis

To investigate the mechanism of XHP's anti-prostate cancer effects, we performed mRNA sequencing on tumor tissues from both groups. We used DESeq for differential gene expression analysis, screening for differentially expressed genes with the following criteria: expression difference fold |log2FoldChange| > 1, significance P-value <0.05. Compared to the control group, 65 differentially regulated mRNAs were identified in the XHP group's tumor tissues, with 9 upregulated and 56 downregulated (Fig. 3A). Differentially expressed miRNAs in each group are listed in Supplementary Material 5. A volcano plot of differentially expressed genes was generated using the R ggplots2 package (Fig. 3B). We employed the R Pheatmap package for bidirectional clustering analysis of the union of differentially expressed genes and samples, using the Euclidean method for distance calculation and complete linkage for clustering. Results are shown in Fig. 3C. Our findings indicate that XHP selectively inhibits the expression of 56 multiply regulated mRNAs and specifically upregulates the expression of 9 mRNAs, providing valuable insights into the mechanisms underlying XHP's anti-prostate cancer effects.

**Fig. 3:**
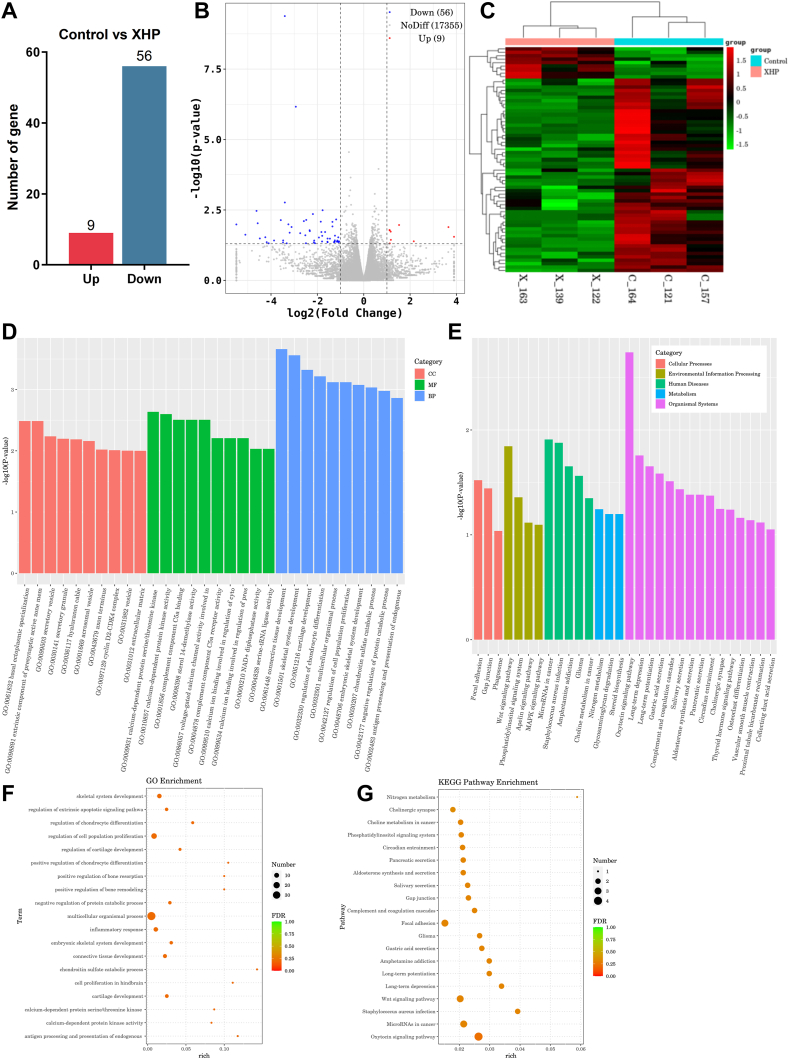
mRNA Sequencing Results and Bioinformatics Analysis. (A) XHP elicits suppression of 56 multiply regulated mRNAs while selectively upregulating 9 mRNAs. (B)Enhanced and diminished expression of distinctively regulated genes (DEGs) highlighted in red and blue, respectively. Criteria: |logFC| ≥1 and adjP ≤0.05. (C)Heatmap analysis depicting differential miRNA expression across each group. Each row corresponds to a distinct miRNA. The color spectrum ranges from red denoting higher expression to green indicating lower expression. (D) Gene Ontology (GO) enrichment analysis of DEGs, categorized by molecular function (MF), biological process (BP), and cellular component (CC). (E) The 20 most significant pathways based on the smallest p-values, reflecting pronounced enrichment.(F–G) Top 20 biological processes and all molecular functions exhibiting enrichment in gene ontology analysis. The x-axis indicates gene ratio, representing the ratio of DEGs to total genes. Dot size is proportional to the gene ratio, and dot color transition from green to orange signifies decreasing adjusted P value.

To delve deeper into the functional classification of XHP-regulated target genes and their associated pathways, we conducted a GO enrichment analysis on differentially expressed genes, categorizing them based on molecular function (MF), biological process (BP), and cellular component (CC) (Fig. 3D). The top 20 GO Term entries with the lowest FDR values, signifying the most notable enrichment, were selected for presentation (Fig. 3F). Our findings reveal that the primary enriched biological functions include multicellular organismal processes, regulation of cell population proliferation, and inflammatory response. This analysis demonstrates that XHP treatment leads to substantial shifts in tumor-associated regulatory functions. Based on the KEGG enrichment analysis of differentially expressed genes, we selected the top 20 Pathways with the smallest p-values, indicating the most significant enrichment, for display (Fig. 3E). We selected the top 20 KEGG pathways with the smallest FDR values for display (Fig. 3G). Results indicate the main enriched pathways are Focal adhesion, Cholinergic synapse, and Wnt signaling pathway. The KEGG enrichment results indicate that in the Wnt signaling pathway, downstream targets CCND2, PRKCG, and CCN4 were found to be downregulated in the XHP intervention group (please refer to Supplementary Material 6 for details). The Wnt signaling pathway plays a crucial role in cellular development and differentiation, participating in various biological processes, such as apoptosis, proliferation, migration, and differentiation. Aberrant activation of the Wnt pathway is closely related to the development of various cancers, including prostate cancer. Intriguingly, the activation of the Wnt pathway can result in the reprogramming of cellular metabolism, thus promoting tumor proliferation. Consequently, the Wnt pathway may be a promising potential therapeutic target for prostate cancer.

### 3.5 XHP modulates the expression of Wnt Pathway-Related Genes and Proteins

To further validate the effect of XHP on the Wnt pathway, we performed PCR analyses on three downregulated Wnt pathway downstream genes (CCND2, PRKCG, CCN4) found in the sequencing results. We discovered that, compared to the control group, the expression of these genes was notably downregulated in tumor tissues following XHP intervention. In vitro experiments revealed a similar trend for both PC-3 and DU145 cell lines, with statistically significant differences observed after XHP-containing serum treatment (Fig. 4A). These findings indicate that XHP suppresses the expression of Wnt pathway downstream genes, which may be the mechanism underlying its anti-tumor effects.

**Fig. 4:**
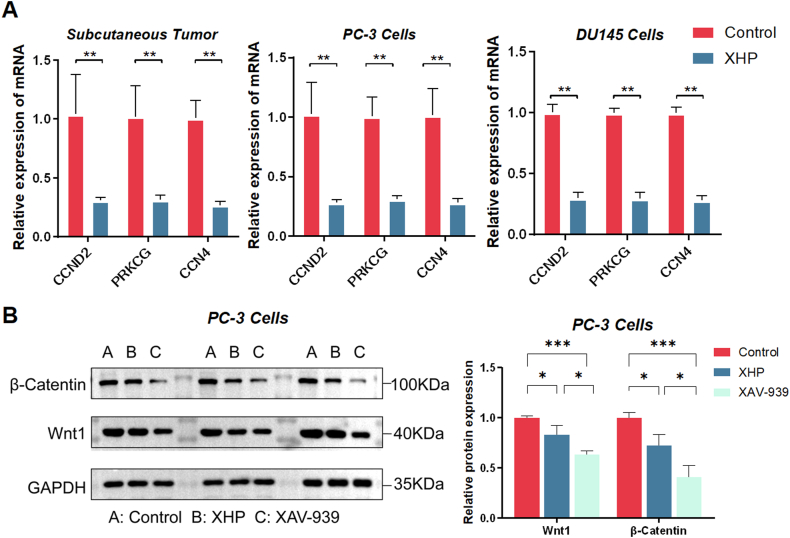
XHP Modulates Expression of Wnt Pathway-Related Genes and Proteins: (A) XHP curtailed mRNA expression of downstream genes (CCND2, PRKCG, CCN4) in tumor tissues, as well as PC-3 and DU145 cells. (B) In PC3 cells, both XHP and XAV-939 suppressed protein expression of β-Catenin and Wnt1. *P* values: *P < 0.05, **P < 0.01, ***P < 0.001.

To further confirm that XHP participates in the mechanism of prostate cancer cell proliferation by modulating Wnt signaling, XAV-939 (a Wnt signaling inhibitor that functions by stabilizing Axin2) was used as a positive control drug [18]. According to the literature, PC3 cells were treated with either blank serum, 10 % XHP-containing serum, or XAV-939 (1 μM) for 24 h. As shown in Fig. 4B, compared to the control group, both XHP and XAV-939 exhibited significant inhibitory effects on the expression of β-Catenin and Wnt1 proteins in PC3 cells (Fig. 4B). This study suggests that XHP might act as a Wnt inhibitor, affecting the expression of β-Catenin and Wnt1 proteins to modulate prostate cancer cell activity.

### 3.6 Glycolytic Enzymes and Glucose Transporters

Metabolic reprogramming plays a crucial role in prostate cancer progression, with a significant interconnection between the Wnt pathway and cellular metabolic reprogramming. In light of this, our investigation aimed to determine the potential of XHP in mitigating the Warburg effect in prostate cancer. Using the 2-NBDG Glucose Uptake Assay Kit, we assessed whether XHP treatment could impede glucose uptake.

The results demonstrated a noteworthy reduction in glucose uptake in prostate tumor tissues treated with XHP, compared to the control group. Similarly, the glucose uptake rates in PC-3 and DU145 cells treated with XHP-containing serum exhibited a significant decrease relative to the control group (Fig. 5A). To ascertain whether XHP also have an effect on normal prostate tissue, we investigated their impact on RWPE-1 cells. The glucose uptake levels in RWPE-1 were notably lower than those observed in tumor cells, and post-XHP treatment, no discernible alteration in glucose uptake capacity was observed. Under simulated hypoxic conditions, PC-3 cells exhibited heightened glucose uptake capabilities. However, following intervention with XHP, a notable downregulation in glucose uptake rates was observed, affirming the regulatory prowess of XHP in glycolysis under hypoxic conditions(Supplementary Material 7). Furthermore, we measured lactate levels in tumor tissues and in vitro cells. Lactate levels in XHP-intervened tumor tissues were markedly lower than those in the control group. Similarly, lactate levels in PC-3 and DU145 cells exhibited a significant reduction 24 h post-intervention with XHP-containing serum, although an opposing trend emerged after 48 h (Fig. 5B). We postulate that this phenomenon may be linked to lactate accumulation following XHP-induced cancer cell apoptosis. Lactate production in glycolysis initiates with glucose uptake and involves a series of catalytic steps. Therefore, we investigated the impact of XHP intervention on the protein levels of key glycolysis-related enzymes, including HIF-1α, GLUT1, HK2, and PKM2. Subsequent to XHP treatment, the protein expression levels of HIF-1α, GLUT1, HK2, and PKM2 in both prostate cancer tissues and the two cell lines showed a significant reduction (Fig. 5C). This suggests that XHP may attenuate the Warburg effect in prostate cancer cells by influencing metabolic reprogramming, thereby inhibiting cell proliferation. We further observed the expression patterns of critical glycolysis-related enzyme proteins under hypoxic and normoxic conditions, as well as the impact of XHP on the expression of glycolysis-related enzyme proteins under both conditions. The findings revealed that under hypoxic conditions, the expression of glycolysis-related enzyme proteins HIF-1α, GLUT1, and PKM2 in PC3 cells were upregulated, indicating an elevation in glycolysis under hypoxia. Interestingly, XHP treatment led to a downregulation of glycolysis-related enzyme proteins under both normoxic and hypoxic conditions, suggesting that XHP can ameliorate the glycolytic phenotype of tumors regardless of oxygen availability(Supplementary Material 7).

**Fig. 5:**
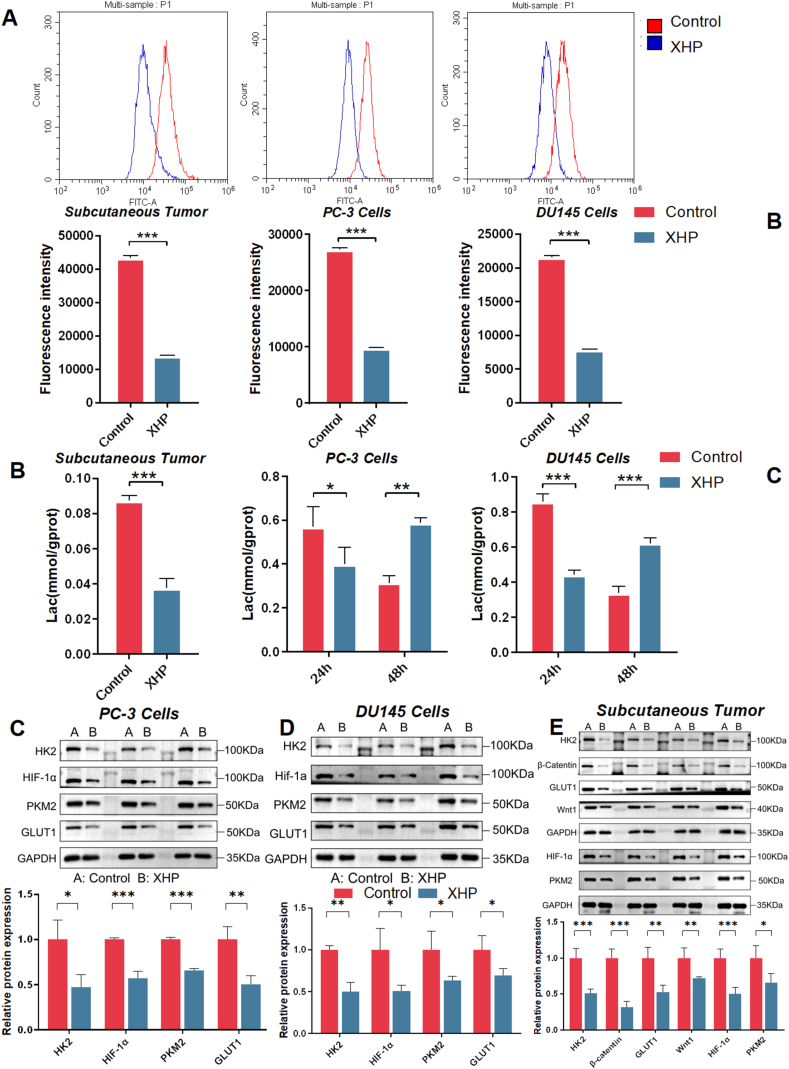
Impact of XHP on Glycolytic Enzymes and Glucose Transporters. (A) XHP intervention suppressed glucose uptake in tumor tissues, as well as PC-3 and DU145 cells. (B) XHP intervention curtailed lactate levels in tumor tissues, as well as PC-3 and DU145 cells. (C) XHP modulated protein levels of various glycolysis-associated enzymes, including HIF-1α, GLUT1, HK2, and PKM2. *P* values: *P < 0.05, **P < 0.01, ***P < 0.001.

### 3.7 Molecular docking of active components of XHP with Wnt1

To further elucidate how the active ingredients in XHP regulate the Wnt pathway and influence prostate cancer proliferation, we conducted molecular docking using LC-MS/MS-identified active compounds as ligands and the key target protein, Wnt1, as the receptor. Docking results indicated favorable binding activity between Wnt1 and the active compounds Glycocholic acid, Glycochenodeoxycholic acid, D-9-AnthrylaAlanine, Cholic acid, Broussonin B, and Abietic acid (Fig. 6A–F). Notably, our findings revealed that the binding energies of these six active components are −8.7, −8.0, −7.5, −7.3, −7.0, and −7.0, respectively, indicating a stable docking interaction. This suggests that these six components derived from XHP may serve as active compounds for intervention in prostate cancer.

**Fig. 6:**
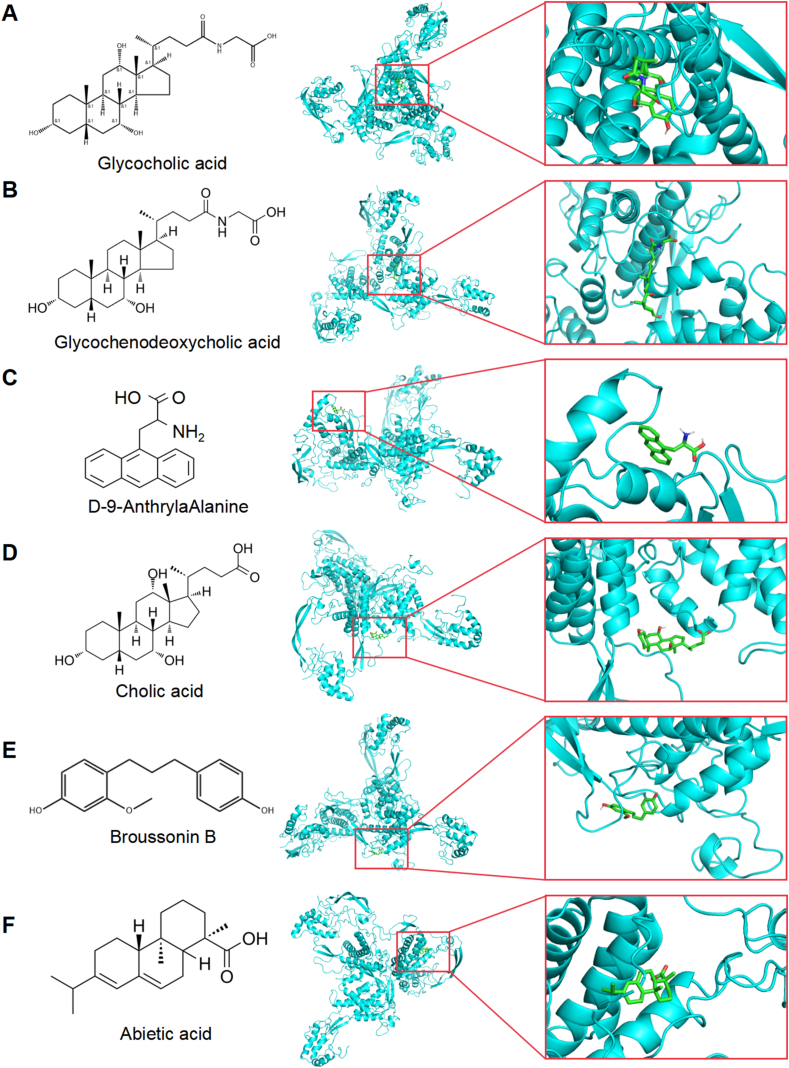
Molecular docking of the active components of XHP (Glycocholic acid, Glycochenodeoxycholic acid, D-9-AnthrylaAlanine, Cholic acid, Broussonin B, Abietic acid) with Wnt1.

## 4 Discussion

Prostate cancer is a disease that increases in incidence with advancing age, heightening susceptibility. As societal advancements and extended life expectancies persist, the prevalence of prostate cancer has shown an upward trajectory [19]. The primary therapeutic modalities for prostate cancer include radical prostatectomy, chemotherapy, androgen deprivation therapy (ADT), and radiotherapy. These interventions aim to enhance the early-stage survival rate for prostate cancer patients [20]. However, a substantial proportion of patients present in advanced metastatic stages, where ADT constitutes the fundamental approach. Despite the initial efficacy of ADT, a significant majority of advanced-stage patients inevitably progress to CRPC [21]. Furthermore, ADT-induced reduction in serum testosterone levels precipitates a spectrum of adverse events, including osteoporosis and cardiovascular ailments, ultimately compromising the patients' quality of life [22]. Hence, there is an imperative need for the development of novel approaches to treat prostate cancer, with the goal of enhancing therapeutic outcomes and reducing mortality rates. Xihuang Pills (XHP), a renowned anticancer prescription originating from China, find widespread application in treating various malignancies, including breast and lung cancers. In previous investigations, we have confirmed XHP's inhibition of prostate cancer cell proliferation; however, the precise underlying mechanisms remain unclear. Consequently, in this study, we comprehensively explore XHP's potential anticancer effects in both in vivo and in vitro settings. Through mRNA sequencing and bioinformatics analyses, we unveil the intricate mechanistic landscape underlying XHP's role in suppressing prostate cancer growth and progression, with a particular focus on the prism of metabolic reprogramming. These findings provide invaluable insights into XHP's therapeutic potential in prostate cancer treatment.

XHP are a composite formulation composed of four traditional Chinese medicinal components: *Calculus Bovis, Moschus, Olibanum, and Myrrha*. Due to this complex composition, there is a need for a more comprehensive identification of the active constituents that contribute to its efficacy. Using LC-MS/MS, we have identified a total of 58 compounds within the XHP extract, including Ketone Huanglian, D-9-AnthrylaAlanine, and Curcumone. This identification emphasizes the intricate nature of XHP's composition, where the combination of various bioactive elements forms the basis for exploring its multifaceted antitumor effects across diverse targets.

Our in vivo experiments, utilizing a subcutaneous xenograft model of PC3 prostate cancer cells, have compellingly demonstrated that XHP treatment significantly inhibits tumor growth without eliciting any discernible alteration in body weight. Immunofluorescence analysis has highlighted XHP's efficacy in effectively suppressing the expression of the proliferative marker Ki67, thereby underscoring its role in inhibiting cellular proliferation. Moreover, the distinct induction of cell apoptosis by XHP is evident, depicting an escalated apoptotic rate accompanied by conspicuous apoptotic body formation. These findings emphasize the potential of XHP in orchestrating the apoptotic pathway and modulating cellular growth control mechanisms.

Subsequent in vitro investigations utilizing XHP-containing serum and extracts have illuminated a concentration-dependent inhibition of PC-3 cell viability. Notably, XHP has exhibited pronounced efficacy in inhibiting the viability of docetaxel-resistant androgen-independent prostate cancer cell lines (PC-3 and DU145). This underscores its potential as a therapeutic avenue against castration-resistant prostate cancer. Furthermore, XHP's capacity to modulate cell cycle progression and inhibit migration and invasion capabilities underscores its multifaceted potential as an antitumor agent. Intriguingly, under simulated hypoxic conditions in vitro, XHP continues to demonstrate remarkable antiproliferative and anti-metastatic capabilities. Hypoxia, a physiological characteristic of tumors, serves as a crucial driver of cancer progression. This underscores the potential of XHP in mitigating the enhanced tumor proliferation and invasion induced by hypoxia.

To elucidate the mechanistic basis of XHP's anti-prostate cancer effects, we conducted mRNA sequencing and pathway analysis, revealing substantial alterations in the gene expression profile following XHP treatment. Enrichment analysis underscored shifts in biological functions and pathways, encompassing multicellular organism processes, regulation of cell population proliferation, inflammatory response, and predominantly, changes associated with the Wnt signaling pathway. Aberrant activation of the Wnt signaling pathway in prostate cancer cells has been extensively documented [23]. Upstream factors of the Wnt pathway, such as Wnt ligands, Frizzled receptors, LRP5/6 co-receptors, exhibit specific alterations in expression within prostate cancer tissues, correlated with tumor differentiation, invasiveness, and prognosis [24]. Conversely, downstream factors of the Wnt pathway, including β-catenin, TCF/LEF transcription factors, and target genes like c-myc and cyclin D1, participate in malignant phenotypes such as cell proliferation, anti-apoptosis, angiogenesis, and metastasis in prostate cancer [25,26]. Our study emphasizes the involvement of the Wnt pathway in prostate cancer development and its potential as a therapeutic target.

We further substantiated XHP's influence on downstream genes of the Wnt pathway (CCND2, PRKCG, CCN4) through in vitro and in vivo experiments. Our results indicate that XHP suppresses the expression of these downstream genes, potentially mediating its anti-tumor effects. Among these, CCND2, a member of the cyclin family regulating various cancer progressions, including prostate cancer, has garnered attention [27]. The protein level of CCND2 is elevated in various prostate cancer cell lines [28]. MiR-615 overexpression has been shown to suppress CCND2 mRNA and protein levels, inhibiting tumor progression, and restoration of Cyclin D2 expression partially reversed miR-615's inhibitory effects on prostate cancer cell proliferation, migration, and invasion [29]. PRKCG, a member of the serine/threonine kinase family, plays a pivotal role in cellular signaling pathways, contributing to tumor formation and progression [30]. Elevated expression in prostate cancer samples correlates with cell proliferation, apoptosis, and cellular motility [31,32]. CCN4, also known as Wnt-1 Induced Signaling Pathway Protein-1 (WISP-1), belongs to the Connective Tissue Growth Factor (CTGF) family [33]. CCN4 is strongly associated with tumor progression and malignancy, promoting cell proliferation, adhesion, migration, invasion, and epithelial-mesenchymal transition via specific signaling pathways [34,35]. Osteoblast-secreted CCN4 participates in prostate cancer bone metastasis through the VCAM-1/integrin α4β1 system [36]. XHP intervention resulted in downregulation of these tumor suppressors (CCND2, PRKCG, CCN4) in tumor tissues and PC-3, DU145 cell lines, revealing XHP's regulatory role in the Wnt pathway. To further substantiate XHP's potential as a Wnt pathway inhibitor, we employed XAV-939, a Wnt signal inhibitor acting by stabilizing Axin2 [18], as a positive control drug. The inhibitory effects of XHP and XAV-939 on the expression of β-Catenin and Wnt1 proteins align with their potential as Wnt pathway inhibitors.

Altered metabolism stands as a distinctive hallmark of cancer. Tumor cells, driven by rapid proliferation, demand heightened energy compared to their normal counterparts [37]. Regardless of oxygen availability, tumor cells exhibit a preference for aerobic glycolysis, augmenting the pace of energy production per glucose unit and yielding surplus intermediates like lactate and acetyl-CoA, favoring rapid cancer cell growth [38]. Moreover, the acidic microenvironment further facilitates polarization of tumor-associated macrophages (TAM), enhancing tumor cell migration and invasion capacity [39]. Unlike other solid tumors, PCa exhibits distinct metabolic patterns throughout its various stages. Healthy prostate epithelial cells primarily rely on citrate oxidation within the Krebs cycle [40]. In early-stage PCa, minimal uptake of 18 F-fluorodeoxyglucose (FDG) is observed, indicating a preference for non-glucose substrates to fulfill anabolic requirements [41]. Studies suggest that early PCa cells prefer lipids and alternative energy sources, leaning more towards oxidative phosphorylation (OXPHOS) rather than glycolysis [42]. However, reports consistently point to a metabolic shift in advanced PCa, especially in aggressive, metastatic, castration-resistant cases [43,44]. Mechanisms involved in the development of CRPC are diverse, encompassing AR gene amplification, overexpression, and mutations leading to constitutive or promiscuous activity [45,46]. These genetic changes induce a metabolic phenotype shift. Over recent decades, research has demonstrated the AR's influence on genes regulating various metabolic processes [14], including glycolysis, leading to metabolic reprogramming in CRPC [47]. 1H NMR analysis reveals a notable shift towards glycolysis in CRPC patients compared to those with benign prostatic hyperplasia or hormone-sensitive PCa [48]. Comparable metabolic alterations have been observed in both human and mouse models of CRPC [49]. These findings implicate the increasing significance of the Warburg effect as a metabolic pathway in CRPC due to multiple metabolic shifts. Notably, our previous predictions and validations of XHP's potential role within the Wnt pathway are closely intertwined with aerobic glycolysis. Activated Wnt/β-catenin signaling enhances glycolytic activity by catalyzing pyruvate dehydrogenase to convert pyruvate to acetyl-CoA, thereby accelerating glucose metabolism [50,51]. This augmentation intensifies cancer cell invasiveness. The disruption of Wnt/β-catenin signaling could downregulate aerobic glycolysis [52]. Furthermore, Wnt/β-catenin pathway activation stimulates tumor glycolysis, propelling macrophage polarization in the microenvironment, leading to epithelial-mesenchymal transition (EMT) development and tumor progression. Silencing Wnt2b or β-catenin inhibits cancer cell glycolysis and EMT [53]. This prompts us to hypothesize that XHP might influence prostate cancer's metabolic reprogramming through its impact on the Wnt pathway. Our observations reveal reduced glucose uptake and lactate production within prostate tumor tissues under XHP intervention, accompanied by a decline in the expression of glycolytic enzymes (GLUT1, HK2, and PKM2). Under both hypoxic and normoxic conditions, XHP consistently exhibits robust inhibitory activity against the expression of glycolysis-related enzymes induced by hypoxia. Notably, XHP does not alter the glycolytic profile of normal prostate epithelial cells. In the glycolytic process of tumor cells, glucose transporters (GLUTs) mediate glucose uptake by transporting glucose directly into tumor cells [[54], [55], [56], [57]]. GLUT 1, located on the cell membrane, is a glucose transporter protein essential for regulating intracellular glycolytic flux. Numerous carcinogenic pathways are associated with the regulation of GLUT1 [56]. Hexokinase 2 (HK2) and pyruvate kinase M2 (PKM2) are key enzymes involved in glucose breakdown and metabolism. Reduced HK2 expression or using HK2 inhibitors underpins increased inhibition of aerobic glycolysis in animal models, restraining tumor proliferation, migration, and metastasis [58]. The final step in glycolytic pathway involves pyruvate kinase muscle (PKM), catalyzing an irreversible reaction. Phosphorylated PKM2 dimers promote rapid nucleotide and amino acid biosynthesis, sustaining the Warburg effect, and facilitating efficient energy supply to tumor cells [59]. PKM2 also associates with metabolic changes driving the transition from benign to malignant phenotypes [57]. The tumor microenvironment is characterized by hypoxia, leading to vascular endothelial growth factor activation and vascular abnormal proliferation under low oxygen conditions. Additionally, localized normoxic cells lose physiological function, and hypoxia drives glucose towards glycolysis, creating a malignant loop [60]. Hypoxia-inducible factor-1 (HIF-1) mediates the adaptive response of tumors to hypoxia. In normoxic conditions, prolyl hydroxylase modifies HIF-1α protein, prompting ubiquitin-proteasome system degradation [61]. Under hypoxia, this process is inhibited. Through HIF-1α pathway mediation, tumor cells upregulate genes associated with glycolysis, energy metabolism, angiogenesis, tumor cell survival, and red blood cell production, including vascular endothelial growth factor (VEGF), GLUTs, and other glycolytic enzymes [62]. This promotes the Warburg effect, fostering multiple malignant phenotypes under hypoxia. HIF-1α is a target of β-catenin, and nuclear β-catenin cooperates with HIF-1α to regulate its transcriptional activity [[63], [64], [65]]. HIF-1α serves as a target of β-catenin, wherein nuclear β-catenin collaborates with HIF-1α to modulate its transcriptional activity [66]. β-catenin's involvement in the cytoplasm-to-nucleus translocation and stabilization of HIF-1α supports metabolic reprogramming induced by Wnt/β-catenin signaling [67]. So, we propose that XHP potentially suppresses the Wnt pathway, downregulates β-catenin expression, consequently dampening HIF-1α expression and stability, ultimately reducing glycolytic enzyme expression. This leads to the inhibition of the Warburg effect, influencing prostate cancer's metabolic reprogramming, thereby impacting tumor proliferation.

XHP presents a promising avenue for combination therapy in PCa through its diverse bioactive compounds and related pharmacological pathways, targeting multiple cellular and molecular target. Based on the identification of 58 compounds in XHP using high-performance liquid chromatography, molecular docking was conducted with the Wnt1 target. The results demonstrated potential interactions between XHP's active components, such as Glycocholic acid, Glycochenodeoxycholic acid, and D-9-AnthrylaAlanine, and the Wnt1 protein. Strong binding activities suggest that these compounds may play a pivotal role in modulating the Wnt pathway and influencing prostate cancer proliferation. Wnt1 stands as a key protein in the Wnt signaling pathway, functioning as a member of the Wnt family [68]. Interacting with cell membrane Frizzled receptors, it initiates a cascade of signaling events [69]. This intracellular signaling activates multiple proteins, including β-Catenin, which is pivotal in cell adhesion, intercellular signaling, and gene transcription regulation [70]. Normally, β-Catenin concentration is tightly controlled within cells through complex mechanisms [71]. However, in tumor tissues, Wnt signaling is activated, leading to β-Catenin accumulation upon Wnt1 binding with Frizzled receptors. Accumulated β-Catenin translocates to the nucleus, binds with the LEF/TCF transcription factor, and facilitates the transcription of specific genes [72]. These genes encode proteins involved in tumor cell proliferation, differentiation, and development [73]. Our study unveils that XHP's active constituents can bind to Wnt1, downregulating Wnt1 expression and activity.

## 5 Conclusion

In conclusion, our investigation has illuminated the multifaceted potential of XHP as a therapeutic agent in prostate cancer. The intricate interplay between XHP's bioactive compounds and pharmacological pathways underscores its promise in providing a multifunctional approach to prostate cancer treatment. The ability of XHP to inhibit tumor growth, proliferation, apoptosis, migration, and invasion collectively highlights its capacity to target key aspects of cancer progression. Moreover, our findings present compelling evidence for XHP's modulation of the Wnt signaling pathway, a pivotal regulator of cell behavior and tumor development. By demonstrating the interaction between XHP's active constituents and Wnt1, we reveal a potential avenue through which XHP exerts its effects. The downregulation of Wnt1 expression and its downstream factors, including β-Catenin/HIF-1α-mediated glycolysis, indicates the intricate mechanism by which XHP disrupts cancer cell metabolism and progression. These insights into XHP's therapeutic potential not only contribute to our understanding of its mode of action but also highlight its promise as a valuable addition to the arsenal of prostate cancer treatments. Further elucidating the intricate details of XHP's effects and its interactions with cellular pathways could pave the way for innovative therapeutic strategies and enhance the clinical management of prostate cancer.

## Data availability statement

Our RNA Sequencing data have been submitted to GEO database(PRJNA1085851). The original contributions presented in the study are included in the article/supplementary material, further inquiries can be directed to the corresponding author.

## Ethics statement

This study was reviewed and approved by Shenzhen Glorybay Biotech Laboratory, with the approval number: [No. RW-IACUC-22-0025].

## Funding

This work was supported by the 10.13039/501100001809National Natural Science Foundation of China Regional Joint Innovation Project (U20A20408), the Youth Project of 10.13039/501100001809National Natural Science Foundation of China (82104861), the 10.13039/501100004735Natural Science Foundation of Hunan Province of China(2023JJ30478), the Bao'an Science and Technology Innovation department of Shenzhen city of China(2021JD068), the Key Projects of First-class Discipline of Integrated Tranditional Chinese and Western Medicine of 10.13039/501100003824Hunan University of Traditional Chinese Medicine(2020zxyjh041).

## CRediT authorship contribution statement

**Fengxia Lin:** Writing – review & editing, Writing – original draft, Methodology, Data curation, Conceptualization. **Yan Long:** Validation, Data curation. **Mingyue Li:** Project administration, Formal analysis, Data curation. **Changlong Cai:** Investigation, Data curation. **Yongrong Wu:** Investigation, Data curation. **Xujun You:** Visualization, Validation, Data curation. **Xuefei Tian:** Writing – review & editing, Conceptualization. **Qing Zhou:** Writing – review & editing, Project administration, Conceptualization.

## Declaration of competing interest

The authors declare that they have no known competing financial interests or personal relationships that could have appeared to influence the work reported in this paper.

## Figures and Tables

**figs1:**
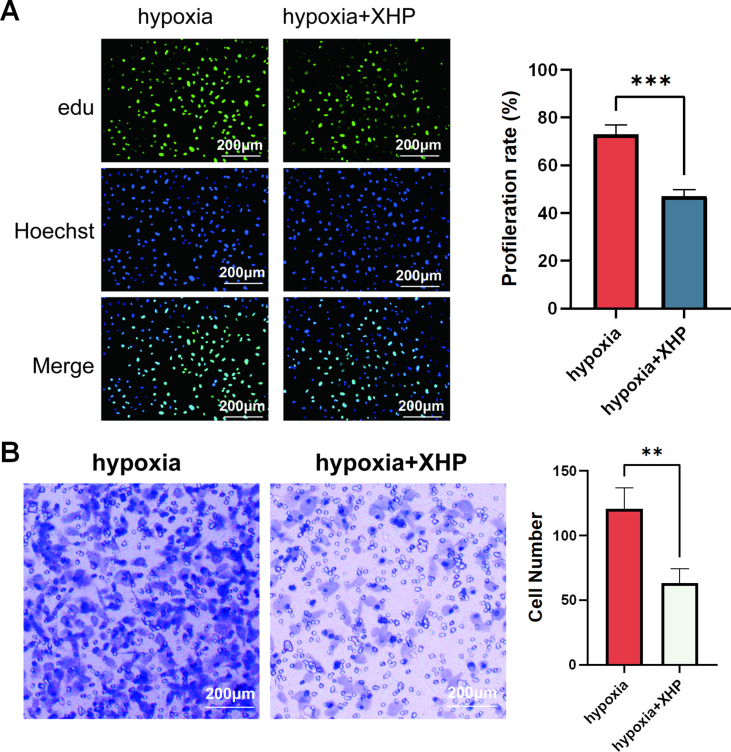


**figs2:**
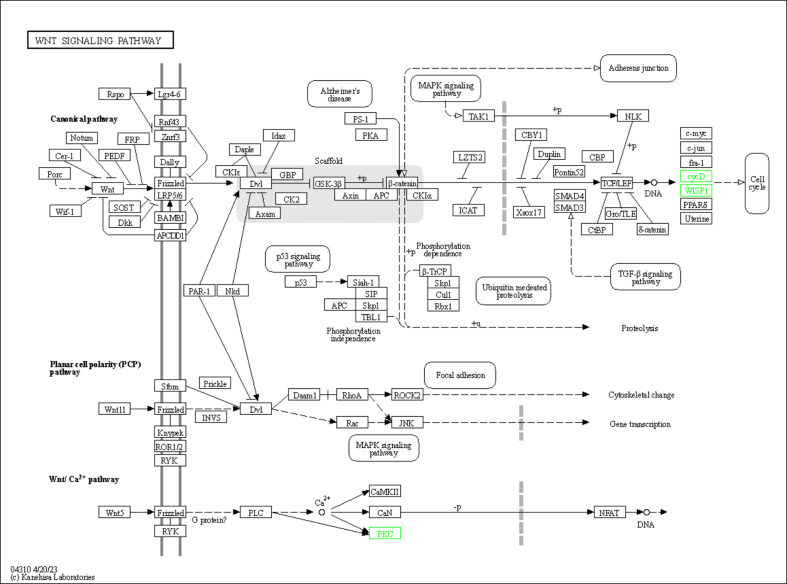


**figs3:**
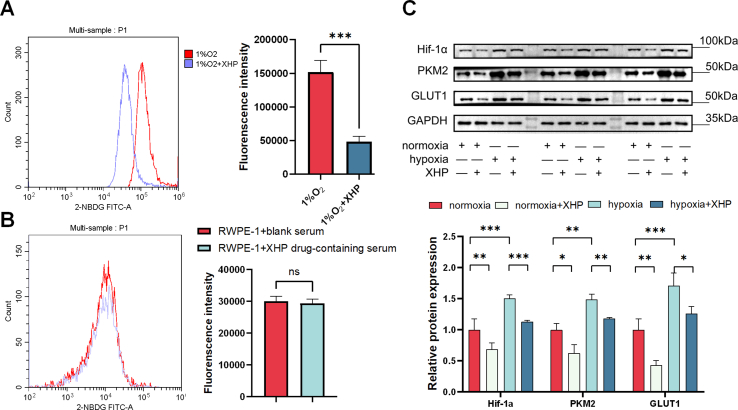


**figs4:**
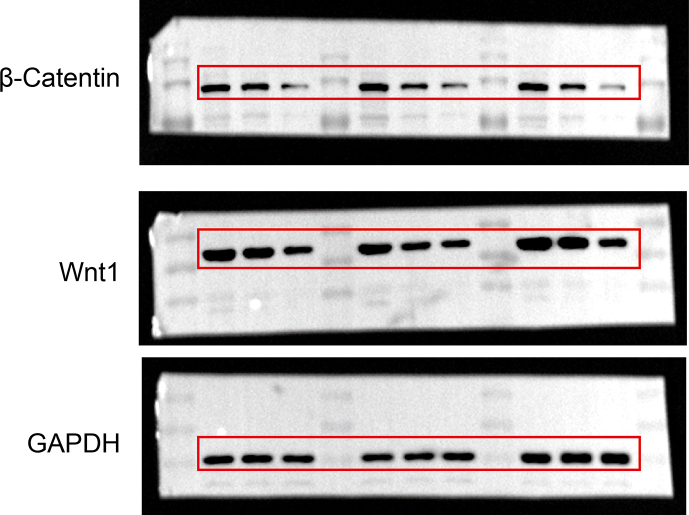


**figs5:**
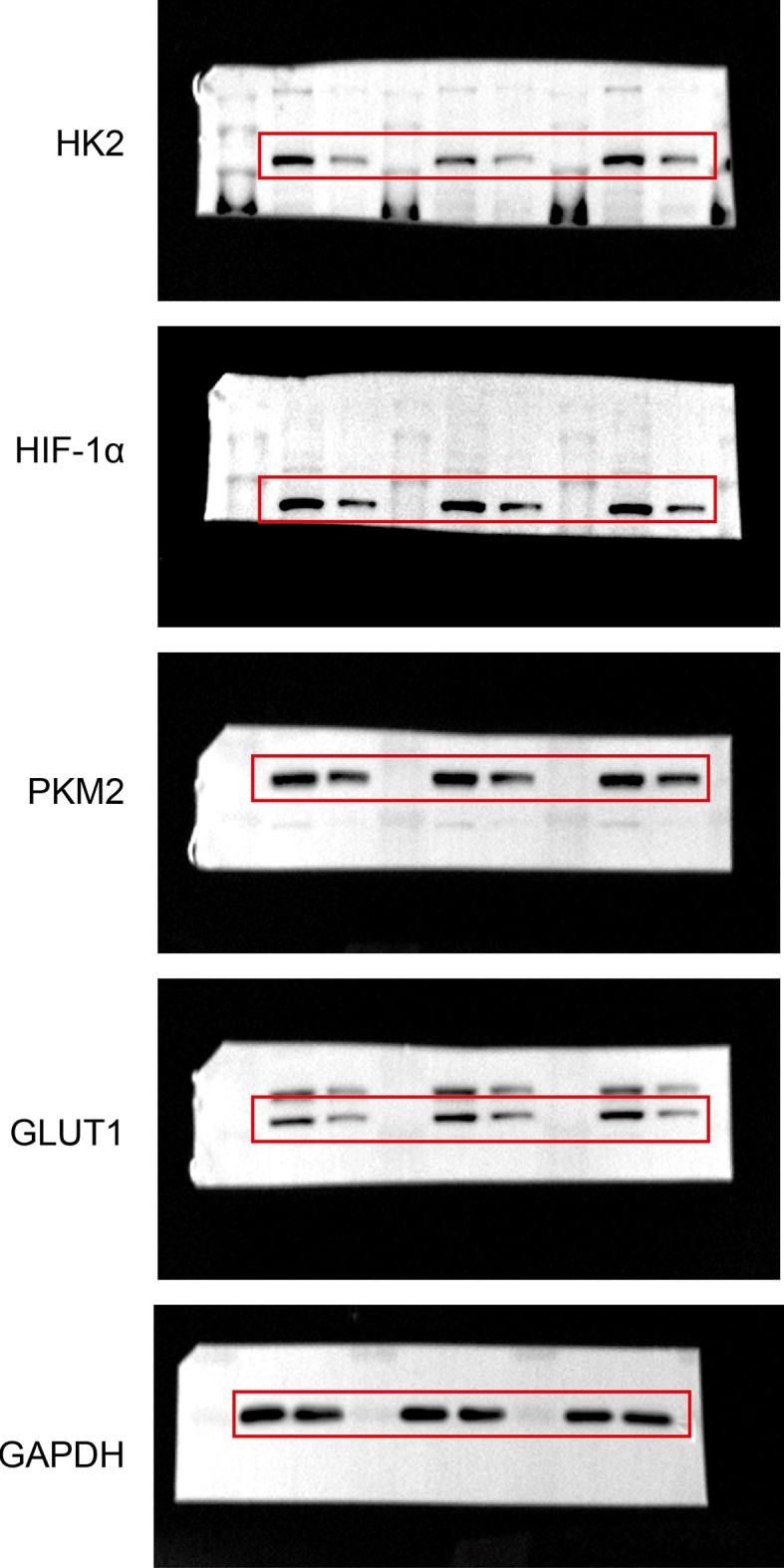


**figs6:**
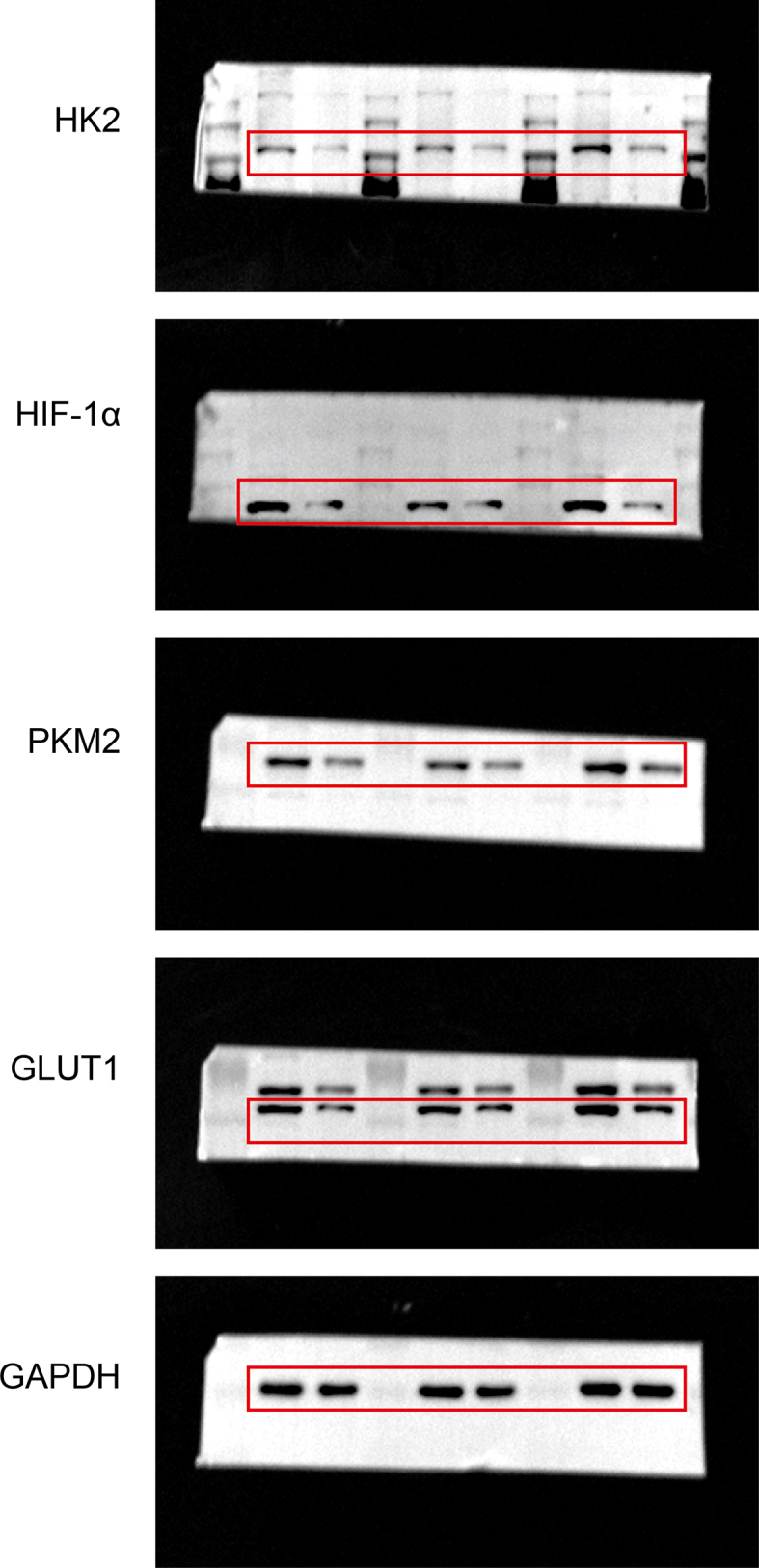


**figs7:**
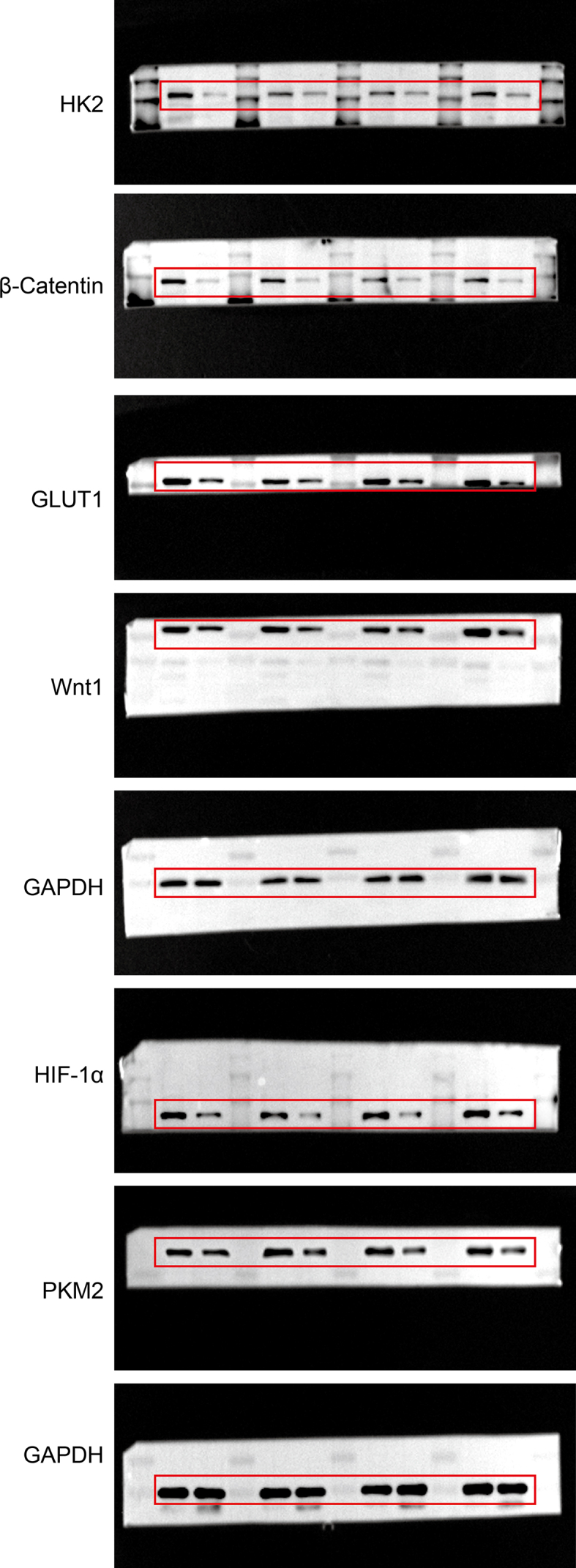


**figs8:**
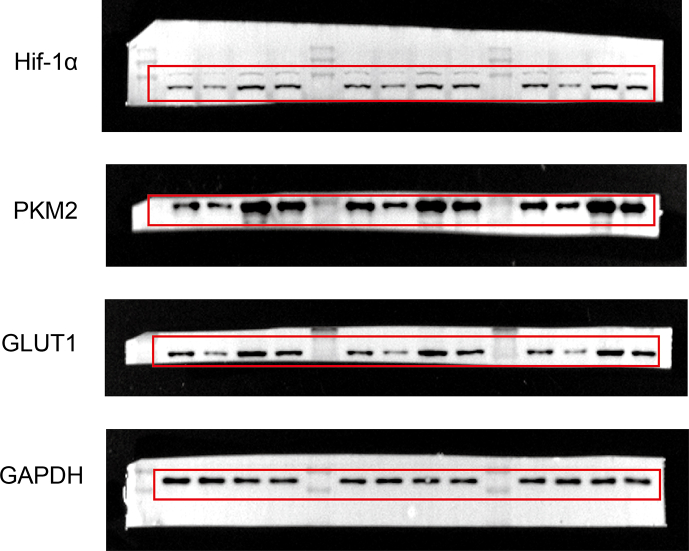


## List of abbreviations

PCa: Prostate cancer
XHP: Xihuang Pills
CRPC: Castration-resistant prostate cancer
CCK-8: Cell Counting Kit-8
QRT-PCR: Real-time quantitative reverse transcription PCR
RNA-seq: Transcriptome from RNA-sequencing
ADT: Androgen deprivation therapy
LC-MS/MS: Liquid Chromatography-Tandem Mass Spectrometry
H&E: Hematoxylin and eosin
GLUTs: Glucose transporters
EMT: Epithelial-mesenchymal transition
PKM: Pyruvate kinase muscle
HIF-1: Hypoxia-inducible factor-1
Wnt: Wingless/Integrated
