# Corrigendum to “Xihuang pills targeting the Warburg effect through inhibition of the Wnt/β-catenin pathway in prostate cancer” [Heliyon Volume 10, Issue 12, June 2024, Article e32914]

**DOI:** 10.1016/j.heliyon.2025.e43562

**Published:** 2025-07-03

**Authors:** Fengxia Lin, Yan Long, Mingyue Li, Changlong Cai, Yongrong Wu, Xujun You, Xuefei Tian, Qing Zhou

**Affiliations:** a Department of Andrology, The First Affiliated Hospital of Hunan University of Chinese Medicine, Changsha, 410007, Hunan Province, China; b Department of Cardiovascular, Shenzhen Bao'an Chinese Medicine Hospital, Guangzhou University of Chinese Medicine, Shenzhen, 518000, Guangdong Province, China; c Graduate School of Hunan University of Chinese Medicine, Changsha, 410208, Hunan Province, China; d Department of Pharmacy, Shenzhen Bao'an Chinese Medicine Hospital, Guangzhou University of Chinese Medicine, Shenzhen, 518000, Guangdong Province, China; e College of Integrated Chinese and Western Medicine, Hunan University of Chinese Medicine, Changsha, 410208, Hunan Province, China; f Department of Urology, Shenzhen Bao'an Chinese Medicine Hospital, Guangzhou University of Chinese Medicine, Shenzhen, 518000, Guangdong Province, China; g School of Traditional Chinese Medicine, Hunan University of Chinese Medicine, Changsha, 410208, Hunan Province, China; h Department of Andrology, Shenzhen Bao'an Chinese Medicine Hospital, Guangzhou University of Chinese Medicine, Shenzhen, 518000, Guangdong Province, China

In this article, the original figure legend lacks sufficient detail about the experimental conditions and imaging methodology, which could lead to ambiguity in interpretation. The description of the tumor images is also overly simplistic and doesn't clearly differentiate between in vivo imaging and post-excision specimens.

Original version of the Fig.1A is as below.

Fig. 1. In vivo anti-tumor activity of XHP. (A) In vivo bioluminescence images and tumor physical map of tumors in each group after treatment."

The corrected version provides more specific information about: (1) the imaging technique used (IVIS spectral imaging), (2) the time point of imaging (day 21 post-intervention), (3) clear differentiation between in vivo imaging (upper panel) and ex vivo specimens (lower panel), and (4) the comparison groups (control vs XHP-treated).

The correct version of Fig.1 and Fig. 1A panels should be as shown below.

**Figure:**
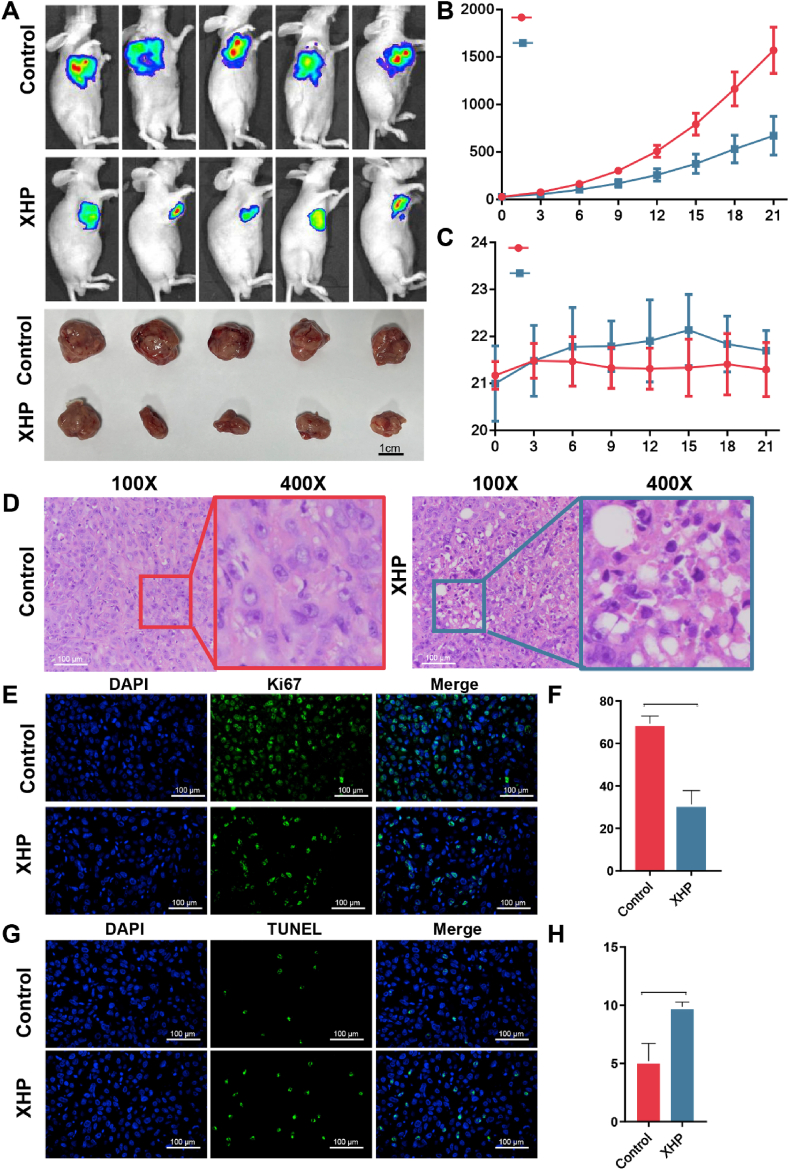

*Fig. 1. In vivo anti-tumor activity of XHP. (A) Upper panel: IVIS spectral imaging of control and XHP-treated mice on day 21 post-intervention; Lower panel: Ex vivo tumor specimens after excision*.

The ***authors*** apologize for the errors.

## Declaration of competing interest

The authors declare that they have no known competing financial interests or personal relationships that could have appeared to influence the work reported in this paper.

