# Supplementary material for: Xihuang pills targeting the Warburg effect through inhibition of the Wnt/β-catenin pathway in prostate cancer
Source: Heliyon. 2024 Jun 15;10(12):e32914. doi: 10.1016/j.heliyon.2024.e32914 (PMC11237975; doi:10.1016/j.heliyon.2024.e32914)
Supplement: Multimedia component 1 [file mmc1.docx]

# Supplement Material 1

| **PC3** |  | **Suspension of XHP extract(mg/L)** | | | | | | | **XHP-containing serum (%)** | | | | | | |
| --- | --- | --- | --- | --- | --- | --- | --- | --- | --- | --- | --- | --- | --- | --- | --- |
|  | **Blank** | | **625** | **125** | **62.5** | **31.25** | **12.5** | **6.25** | **10%** | **2%** | **1%** | **0.5%** | **0.1%** | **0.01%** |  |
| **0H** | **0.481** | | **0.470** | **0.484** | **0.478** | **0.474** | **0.488** | **0.482** | **0.489** | **0.477** | **0.488** | **0.467** | **0.470** | **0.479** |  |
|  | **0.486** | | **0.483** | **0.475** | **0.481** | **0.465** | **0.478** | **0.471** | **0.475** | **0.461** | **0.477** | **0.482** | **0.469** | **0.489** |  |
|  | **0.482** | | **0.465** | **0.477** | **0.463** | **0.475** | **0.475** | **0.478** | **0.471** | **0.471** | **0.477** | **0.489** | **0.484** | **0.467** |  |
| **average** | **0.483** | | **0.473** | **0.479** | **0.474** | **0.471** | **0.480** | **0.477** | **0.478** | **0.470** | **0.481** | **0.479** | **0.474** | **0.478** |  |
| **stdev** | **0.003** | | **0.009** | **0.005** | **0.010** | **0.006** | **0.007** | **0.006** | **0.009** | **0.008** | **0.006** | **0.011** | **0.008** | **0.011** |  |
| **48H** | **1.008** | | **0.585** | **0.685** | **0.710** | **0.811** | **0.897** | **0.941** | **0.738** | **0.841** | **0.863** | **0.906** | **1.035** | **1.065** |  |
|  | **1.035** | | **0.581** | **0.695** | **0.732** | **0.814** | **0.885** | **0.921** | **0.753** | **0.826** | **0.887** | **0.915** | **1.022** | **1.041** |  |
|  | **1.028** | | **0.586** | **0.653** | **0.726** | **0.835** | **0.867** | **0.912** | **0.729** | **0.835** | **0.893** | **0.932** | **1.032** | **1.055** |  |
| **average** | **1.024** | | **0.584** | **0.678** | **0.723** | **0.820** | **0.883** | **0.925** | **0.740** | **0.834** | **0.881** | **0.918** | **1.030** | **1.054** |  |
| **stdev** | **0.014** | | **0.003** | **0.022** | **0.011** | **0.013** | **0.015** | **0.015** | **0.012** | **0.008** | **0.016** | **0.013** | **0.007** | **0.012** |  |
| **Proliferation rate/%** |  | | **-42.95%** | **-33.80%** | **-29.40%** | **-19.90%** | **-13.74%** | **-9.67%** | **-27.71%** | **-18.53%** | **-13.94%** | **-10.35%** | **0.59%** | **2.93%** |  |

# Supplementary Material 2

# Homo sapiens cyclin D2 (CCND2), mRNA.

# NM_001759

Primer F：5’ ACCTTCCGCAGTGCTCCTA3’

Primer R：5’ CCCAGCCAAGAAACGGTCC3’

Pos:110-270

Amplified product: Size: 161 bps

# Homo sapiens protein kinase C, gamma (PRKCG), mRNA.

# NM_002739

Primer F：5’ AGCCACAAGTTCACCGCTC3’

Primer R：5’ GGACACTCGAAGGTCACAAAT3’

Pos:103-257

Amplified product: Size: 155 bps

# Homo sapiens cellular communication network factor 4 (CCN4), transcript variant 1, mRNA

# NM_003882

Primer F：5’ CCAGCCTAACTGCAAGTACAA3’

Primer R：5’ GGCGTCGTCCTCACATACC3’

Pos:408-567

Amplified product: Size: 160 bps

# Homo sapiens glyceraldehyde-3-phosphate dehydrogenase (GAPDH), transcript variant 2, mRNA

# NM_001256799

Primer F：5’ GGAGCGAGATCCCTCCAAAAT 3’

Primer R：5’ GGCTGTTGTCATACTTCTCATGG 3’

Pos: 108-304

Amplified product: Size: 197 bps

# Supplement Material 3


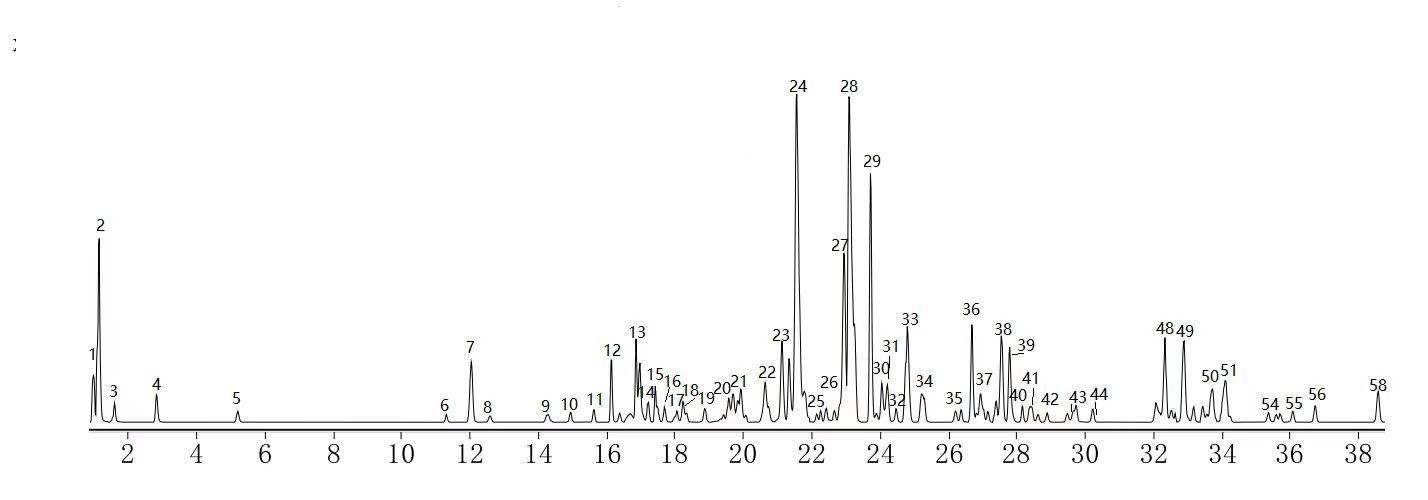


Total ion chromatograms (TIC) from XHP

List of the identified compounds

| Nub | tR/min | PIM | AM | Error | MGF | Mass | Name |
| --- | --- | --- | --- | --- | --- | --- | --- |
| 1 | 1.155 | M+H=705.0853 | M-H=701.1955 | - |  |  |  |
| 2 | 1.602 |  | M-H=355.0366 | -1.48 | C20H8N2O5 | 356.0433 | - |
| 3 | 2.861 |  | M-H=169.0181 | -6.71 | C8H2N4O | 170.0242 | - |
| 4 | 5.213 |  | M-H=153.0278 | 0.11 | C3H8NO6 | 154.0626 | - |
| 5 | 11.308 |  | M-H=633.0767 | -5.05 | C27H22O18 | 4.17 | Kezitanin |
| 6 | 12.307 |  | M+CH3COOH=325.1322 | -0.8 | C17H15NO2 | 256.1103 | D-9-AnthrylaAlanine |
| 7 | 12.584 |  | M-H=295.1214 | 0.29 | C18H18NO3 | 296.1297 | Trimethylolpropane triacrylate |
| 8 | 14.952 |  | M+COOH=309.1448 | 1.28 | C14H20N2O3 | 264.1474 | Subaphyllin |
| 9 | 15.615 |  | M-H=281.1473 | 1.21 | C11H24NO7 | 282.1878 | - |
| 10 | 16.133 | M+H=679.5128 |  |  |  |  |  |
| 11 | 16.878 | M+H=792.5919 |  |  |  |  |  |
| 12 | 17.343 | M+H=263.1257 |  | 1.32 | C15H18O4 | 262.1184 | 1,3,4-Oxadiazol-2(3H)-one |
| 13 | 17.69 | M+Na=195.1364 |  | -1.89 | C10H20O2 | 172.1463 | Octyl acetate |
| 14 | 18.038 | M+H=231.1371 |  | 3.92 | C15H18O2 | 230.1271 | Curzerenone |
| 15 | 18.895 |  | M+CH3COOH=309.1417 | 2.83 | C10H19NO6 | 249.1212 | Ethyl 2-acetamido-2-deoxyhexopyranoside |
| 16 | 19.204 | M+H=247.1319 |  | 4.23 | C15H18O3 | 246.1256 | rel-1S,2S-epoxy-3R-methoxy-4R-furanogermacr-10(15)-en-6-one |
| 17 | 20.520 | M+Na=279.2287 |  | 3.24 | C16H32O2 | 256.2402 | palmitic acid |
| 18 | 20.617 |  | M-H=277.1123 | -5.46 | C18H16NO2 | 278.1181 |  |
| 19 | 21.330 |  | M-H=261.1161 | -11.43 | C15H18O4 | 262.1184 | 1,3,4-Oxadiazol-2(3H)-one |
| 20 | 21.528 |  | M-H=514.2881 | -7.27 | C26H45NO7S | 515.2917 | Taurocholic acid |
| 21 | 22.129 | M+Na=321.2408 |  | -3.56 | C18H34O3 | 298.2508 | Ricinoleic acid |
| 22 | 22.462 | M+H=303.2312 |  | 1.76 | C20H30O2 | 302.2246 | Abietic acid |
| 23 | 22.936 |  | M-H=293.1462 | 0.57 | C10H22N4O6 | - | Ethylenediamine Diaceturate |
| 24 | 23.073 | M+H=429.2410 | M-H=427.2334 | 0.99 | - | - | Rhodomollein III |
| 25 |  | M+H=229.1209 |  | 5.53 | C15H16O2 | 228.115 | Myrrhone |
| 26 | 23.715 | M+H=466.3176 | M-H=464.3094 | 0.13 | C26H43NO6 | 465.309 | Glycocholic acid |
| 27 | 24.791 |  | M-H=498.2995 | -1.23 | C30H43O6 | 499.3060 | - |
| 28 | 25.193 | M+Na=223.1671 |  | -1.27 | C12H24O2 | 200.1776 | Decyl acetate |
| 29 | 25.922 | M+NH4=274.2741 |  | -0.07 | C16H32O2 | 256.2402 | Lauric Acid Isobutyl Ester |
| 30 | 26.303 |  | M+H=229.1214 | 3.98 | C15H16O2 | 228.115 | Myrrhone |
| 31 | 26.696 |  | M+Na=339.2518 | -3.74 | C18H36O4 | 316.2604 | 9,10-Dihydroxystearic acid |
| 32 | 26.684 | M+H=500.3032 | M-H=498.2992 | -0.08 | C26H45NO6S | 499.2968 | taurodeoxycholic acid |
| 33 | 26.701 | M+NH4=426.3198 |  | 4.47 | C24H40O5 | 408.2876 | Cholic acid |
| 34 | 26.981 |  | M-H=501.3300 | -0.51 | C26H48NO8 | 502.3308 |  |
| 35 | 27.541 |  | M-H=331.1995 | 1.47 | C16H30NO6 | 332.2073 | - |
| 36 | 27.79 | M+H=450.3223 | M-H=448.3154 | 0.96 | C26H43NO5 | 449.3141 | Glycochenodeoxycholic acid |
| 37 | 27.91 | M+Na=181.1209 |  | -5.73 | C9H18O2 | 158.1307 | 1-Octanol |
| 38 | 29.135 | M+Na=305.2447 |  | 1.79 | C18H34O2 | 282.2259 | Oleic acid |
| 39 | 29.751 | M+H=303.2314 |  | -0.32 | C20H30O2 | 302.2246 | Abietic acid |
| 40 | 30.225 |  | M+COOH=489.3610 | -4.97 | C29H48O3 | 444.3603 | - |
| 41 | 30.461 | M+H=363.2529 |  | 1.88 | C22H34O4 | 362.2437 | Fritillebic acid |
| 42 | 30.773 | M+H=259.1329 |  | -0.2 | C16H18O3 | 258.1256 | Broussonin B |
| 43 | 31.239 | M+H=213.1271 |  | 0.83 | C15H16O | 212.1201 | [2-(2-phenylethyl)phenyl]methanol |
| 44 | 31.412 | M+H=383.2792 |  | 0.42 | C22H38O5 |  |  |
| 45 | 31.819 | M+H=305.2472 |  | 1.15 | C20H32O2 | 302.2246 | Eicosanetetraenoic acid |
| 46 | 32.328 |  | M-H=391.2936 | 1.14 | C20H42NO6 | 360.3287 | - |
| 47 | 32.981 |  | M-H=487.3519 | -0.2 | C26H50NO7 | 488.3587 | - |
| 48 | 33.703 |  | M-H=441.2715 | -2.91 | C20H42O10 | 282.3287 | - |
| 49 | 34.370 | M+H=231.1365 |  | 5.85 | C15H18O2 | 230.1307 | Curcumone |
| 50 | 34.697 | M+H=229.1225 |  | -0.83 | C15H16O2 | 228.115 | Myrrhone |
| 51 | 35.099 | M+H=323.2583 |  | -0.87 | C20H34O3 | 338.2444 | Glutinic acid |
| 52 | 35.248 | M+H=471.3471 | M-H=469.3372 | -0.08 | C30H46O4 | 470.3396 | 11-Oxo-β-boswellic acid |
| 53 | 35.395 |  | M-H=295.2381 | 3.04 | C17H32N2O2 | 296.2464 | - |
| 54 | 36.076 | M+H=453.2475 | M-H=455.4603 | 1.02 | C24H36O8 | 452.241 | Diacetoxytetrahydroxytaxadiene |
| 55 | 36.718 | M-H=455.3617 |  | -0.06 | C26H50NO5 | 456.3289 | - |
| 56 | 37.981 | M+H=347.2589 |  | -2.06 | C22H34O3 | 346.2513 | Anacardic acid B |
| 57 | 38.556 |  | M-H=467.3211 | -3.26 | C23H48O9 | 468.3756 | - |
| 58 | 38.908 | M+H=513.3575 |  | 1.03 | C32H48O5 | 512.3502 | Acetyl-11-keto-β-boswellic acid |
